# Selected Conference Abstracts from “The Regulation of Proteostasis in Cancer”

**DOI:** 10.1038/s41420-020-0248-5

**Published:** 2020-04-07

**St. Petersburg, Russia, 11-12 October 2019**

http://cancerproteostasis.com/

Organizers: Mauro Piacentini and Nick Barlev

Volume 6 | Supplement 1

This conference was organized by the Institute of Cytology of Russian Academy of Sciences and was supported by the Mega-Grant Program 14.W03.31.0029, launched by the Russian Government in 2010.

**Sponsorship:** Publication of the supplement was sponsored by the Institute of Cytology as part of the fulfilment of the Russian Government Program for the Recruitment of the leading scientists into the Russian Institutions of Higher Education 14.W03.31.0029. All content was reviewed and approved by the organizers, which held full responsibility for the abstract selections.

## RPC 01

### Alteration in gene expression and increase karyotypic instability in chinese hamster cells with different resistance to ethidium bromide after treatment with polyallylamine

#### Larisa L Alekseenko^1^, Mariia A Shilina^1^, Irina V Kozhukharova^1^, Olga G Lyublinskaya^1^, Valentina P. Ivanova^2^, Nikolay N Nikolsky^1^, Tatiana M Grinchuk^1^

##### ^1^Institute of Cytology RAS, St.-Petersburg, Russia; ^2^Institute of Evolutionary Physiology and Biochemistry RAS, St.-Petersburg, Russia

The aim of this work was to analyze the changes in gene expression and karyotype of CHL V-79 RJK cells sensitive and resistant to multidrug resistance agents (MDR), after treatment with the synthetic polymer polyallylamine (PAA). Short-term (1.5 h) exposure to PAA (100 μg/ml) on cells of the CHL V-79 RJK line sensitive to MDR agent, ethidium bromide (BE) and on Vebr-5 cells (CHL V-79 RJK, resistant to 5 ɣ BE) was cytotoxic and, 24 h after exposure, 90% of the cell population died. The single surviving PAA cells have resumed a proliferation after 72 h. Karyological analysis and investigation of gene expression of surviving cells were performed at passage 2 after treatment with PAA. G-banding analysis of the chromosomes showed that CHL V-79 RJK cells had a stable karyotype. Vebr-5 cells were characterized by the presence of a homogeneously stained region (HSR) at 1q26 at the location of the wild-type MDR genes. The length of the HSR varied within certain limits. After PAA treatment, a trend was observed in Vebr-5 cells to increase the HSR length and numerous changes in the structure of the karyotype: the appearance of atypical chromosomes, additional chromosomal copies, and an increase in the variability of the number of chromosomes. The structure of the karyotype CHL V-79 RJK, sensitive to BE, after exposure to PAA, is also characterized by destruction of stability, but to a lesser extent. Analysis of gene expression using Rial-Time PCR showed that mdr1 gene expression increased in the Vebr-5 line, which correlated well with the appearance of HSR on the Z6 chromosome. Enhanced p53 and a reduced top2a gene expression levels in both cell lines after exposure to PAA indicated that damaged cells could remain in the surviving cell population, while cell repair and selection processes were still ongoing. The basic genes expression level of the hsp90, hsc70, and grp78 was significantly higher in the Vebr-5 line. After exposure to PAA, a decrease in the expression of hsp90 and hsc70 was observed. The level of grp78 remained unchanged in both cell lines. Expression of c-fos, which is a transcription factor and is responsible for the proliferation, differentiation, and apoptotic cell death, is significantly enhanced in the Vebr-5 cell line after exposure to PAA, in contrast to BE-sensitive CHL V-79 RJK. The MTT test showed that the Vebr-5 cell line is more resistant to doxorubicin (1-100 µg/ml) than the sensitive line. The treatment of PAA cells did not contribute to the appearance and progression of MDR in the CHL V-79 RJK and Vebr-5 cell lines. In conclusion, the authors demonstrated that short-term treatment of RJK CHL V-79 and Vebr-5 cells with cytotoxic doses of PAA led to karyotypic instability, was accompanied by changes the p53, c-fos, top2a, hsp90 and hsc70 genes expression level, but did not contribute to progression MDR.

Disclosure: The work was supported by the Russian Science Foundation (project 19-14-00108).

## RPC 02

### Polyploidy related induction of morphogenetic signaling is mediated via proteasome pathway

#### Olga V Anatskaya^1^, Jekaterina Erenpreisa^2^, Alessandro Giuliani^3^, Anna S Tsimokha^1^, Kristine Salmina^2^, Alexander E Vinogradov^1^

##### ^1^Institute of Cytology RAS, St.-Petersburg, Russia; ^2^Latvian Biomedical Research and Study Centre, Riga, Latvia; ^3^Istituto Superiore di Sanità, Rome, Italy

The data obtained with tumor genome-wide studies indicate that polyploidy prevails among about 30% human tumors of various localizations. Also, recent studies evidence that polyploidy may increase biological plasticity and induce manifestations of stemness and embryonality thus promoting transformation, tumor progression and drug resistance^1^. Functional implications of polyploidy in tumor initiation and progression as well as in the nature of ploidy-embryonality relationships remain unclear. The objective of the study is to investigate the effect of polyploidy on pathway of morphogenesis and stemness. To identify ploidy associated genes, the authors first applied pair-wise cross-species transcriptome comparison^2,3^ of human and mouse tissues with various degree of polyploidy (i.e. human and mouse heart, liver and placenta) and principal component analysis (PCA) of the same tissues. Than the authors investigated the data with protein interaction network analysis and gene module functional enrichment analysis. Manifestations of stemness were evaluated by statistically significant associations of differentially expressed genes with molecular pathways of the NCBI BioSystems database^5^, containing the names of multi- and pluripotency signaling pathway regulators in the annotation (WNT, NOTCH, HIPPO, TGFb, FGF, FOXO, POU5F1, NANOG, SOX) and terms related to stemming, multi and pluripotency and differentiation. In human and mouse heart and liver, the network of protein-protein interactions for genes encoding multipotency regulators showed that polyploidy is associated with increased manifestations of stemness. It is noteworthy that many hubs of the network are involved in the protein degradation by proteasome that play an important role in the maintaining of stemness and multipotency. By regulating the life span of stem factors, the proteasome system determines the choice of a cell between maintaining a pluripotent state or differentiation^4^. The data presented here promotes understanding of the role of polyploidy in normal organogenesis, regeneration and transformation. Also, the obtained results shed light on the relationships between polyploidy and embryonalization and uncover mechanisms regulating this phenomenon.

References

1. Erenpreisa, J. et al. Stress-induced polyploidy shifts somatic cells towards a pro-tumourogenic unicellular gene transcription network. Cancer Hypotheses 1, 1–20 (2018).

2. Vazquez-Martin, A. et al. Somatic polyploidy is associated with the upregulation of c-MYC interacting genes and EMT-like signature. Oncotarget 7, (2016). 10.18632/oncotarget.12118.

3. Anatskaya, O. V. & Vinogradov, A. E. Somatic polyploidy promotes cell function under stress and energy depletion: evidence from tissue-specific mammal transcriptome. Funct. Integr. Genomics 10, 433–446 (2010). 10.1007/s10142-010-0180-5.

4. Selenina, A. V, Tsimokha, A. S. & Tomilin, A. N. Proteasomes in Protein Homeostasis of Pluripotent Stem Cells. Acta Naturae 9, 39–47.

5. https://www.ncbi.nlm.nih.gov/biosystems/.

## RPC 03

### Autophagy level in lung adenocarcinoma cells with different EGFR status

#### Ekaterina Baidyuk, Oleg Y Shuvalov, Oleg M Semenov, Alexandra A Daks, Olga A Fedorova, Mauro Piacentini, Nickolai A. Barlev

##### ^1^Institute of Cytology RAS, St.-Petersburg, Russia; ^2^Moscow Institute of Physics and Technology (National Research University), Dolgoprudny, Russia

The aim of the work was to assess the autophagy level in the cells of lung adenocarcinoma with different mutations in the EGFR gene, and hence by with varying resistance to tyrosine kinase inhibitors. Using CRISPR/Cas9 gene editing system the authors introduced specific mutations in the 20 and 21 exons of the EGFR gene in H1299 cells either as single (H1299/T790M and H1299/L858R, respectively) or double mutations (H1299/L858R/T790M). Anti-EGFR primary antibodies (Santa Cruz, USA) and fluorescently-labeled secondary antibodies (Invitrogen, USA) were used in for immunocytochemical analysis. To quantify the level of autophagy the Muse Autophagy LC3-antibody based Kit (Merck, Germany) was used. Measurements of the of the cells fluorescence level were carried out using Guava Easycyte 8 Flow Cytometer. Immunofluorescence analysis showed that EGFR display clear bright cellular membrane signal in H1299 WT (wild tipe) and in H1299/T790M cell. In H1299 L858R cells, as well as in cells with double mutations (H1299 L858R/T790M), EGFR was not detected on membrane, or signal was too low to be detected by confocal microscopy. To quantify the level of autophagy the Muse Autophagy LC3-antibody based Kit (Merck, Germany) was used. Measurements of the of the cells fluorescence level were carried out using Guava Easycyte 8 Flow Cytometer. Using this method, the authors showed that the double mutant EGFR H1299 (H1299 L858R/T790M) have a lowest level of autophagy. The level of autophagy in cells with single mutations was also lower than in the wild type. The fluorescence intensity in wild-type EGFR cells and with mutation in 20 exon (T790M) in the experiment increased in 2.5 times, in cells with mutation in 21 exon (L858R) and in cells with double mutation of EGFR gene the level increased 1.7 and 2 times, respectively. Western blot analysis gave the same results. Thus, H1299 cell lines with different EGFR status have a different autophagy levels and H1299/L858R/T790M cells have the lowest.

Disclosure: This study was supported by the Mega grant 14.W03.31.0029 and Russian Science Foundation#19-45-02011

## RPC 04

### A DNA nanostructure platform for drug delivery, miRNA sequestering and cancer proteostasis

#### Silvia Biocca^1^, Alessandro Desideri^2^

##### ^1^Department of Systems Medicine University of Roma Tor Vergata, Rome, Italy; ^2^Department of Biology University of Roma Tor Vergata, Rome, Italy

MicroRNAs (miRNAs) are the family non-coding RNAs that have a crucial role in the regulation of gene expression. Although the complementary region between miRNA and its target is comparatively short it may bind many transcripts, containing the sites for a given miRNA. Thus, one miRNA therefore has the potential to regulate hundreds of different mRNA targets. Currently it is estimated that microRNAs regulate as many as 60% of all human mRNAs. Recently, nanotechnology field has grown fast and an impressive variety of materials has been used for building nanoparticles of different size and shape. In particular DNA, due to its intrinsic biocompatible, nontoxic and stable properties has been extensively investigated for designing programmable, self-assembling nanostructures for various biomedical applications, such as drug delivery, cellular biosensors and in vivo imaging. In the last years, our laboratory has been involved in the characterization of the structure of truncated octahedral DNA nanocages in silico and in vitro^1–7^. DNA nanocages are composed by twelve double helices, each one connected to four single stranded linkers, and are fully covalently bound, making the structure highly stable also in biological fluids. DNA nanocages are not cytotoxic and their intracellular uptake is observed in cells expressing scavenger receptors^8^. When functionalized with folate molecules and tested in αFR over-expressing tumor cell lines, DNA nanocages are very selectively internalized in vesicular structures in the early endosomal system, and can be efficiently loaded with the chemotherapeutic agent doxorubicin for a targeted drug delivery^9,10^. In this presentation, I will show how DNA nanocages can be further functionalized to confer specific properties, such as specific oligonucleotide binding activity, paving the way for their use as selective miRNA sequestering units. The importance of shape, size and of the presence of covalent or non-covalent bonds on the DNA structure, in modulating the receptor-mediated cellular uptake and the cage degradation rate will be also discussed.

References

1. Falconi, M. et al. Deciphering the Structural Properties That Confer Stability to a DNA Nanocage. ACS Nano 3, 1813–1822 (2009). 10.1021/nn900468y.

2. Oliveira, C. L. P. et al. Structure of Nanoscale Truncated Octahedral DNA Cages: Variation of Single-Stranded Linker Regions and Influence on Assembly Yields. ACS Nano 4, 1367–1376 (2010). 10.1021/nn901510v.

3. Juul, S. et al. Temperature-Controlled Encapsulation and Release of an Active Enzyme in the Cavity of a Self-Assembled DNA Nanocage. ACS Nano 7, 9724–9734 (2013). 10.1021/nn4030543.

4. Alves, C. et al. A Simple and Fast Semiautomatic Procedure for the Atomistic Modeling of Complex DNA Polyhedra. J. Chem. Inf. Model. 56, 941–949 (2016). 10.1021/acs.jcim.5b00586.

5. Franch, O. et al. DNA hairpins promote temperature controlled cargo encapsulation in a truncated octahedral nanocage structure family. Nanoscale 8, 13333–13341 (2016). 10.1039/C6NR01806H.

6. Ottaviani, A. et al. Engineering a responsive DNA triple helix into an octahedral DNA nanostructure for a reversible opening/closing switching mechanism: a computational and experimental integrated study. Nucleic Acids Res. 46, 9951–9959 (2018). 10.1093/nar/gky857.

7. Raniolo, S. et al. Cellular uptake of covalent and non-covalent DNA nanostructures with different sizes and geometries. Nanoscale 11, 10808–10818 (2019). 10.1039/C9NR02006C.

8. Vindigni, G. et al. Receptor-Mediated Entry of Pristine Octahedral DNA Nanocages in Mammalian Cells. ACS Nano 10, 5971–5979 (2016). 10.1021/acsnano.6b01402.

9. Raniolo, S. et al. Entry, fate and degradation of DNA nanocages in mammalian cells: a matter of receptors. Nanoscale 10, 12078–12086 (2018). 10.1039/C8NR02411A.

10. Raniolo, S. et al. Selective targeting and degradation of doxorubicin-loaded folate-functionalized DNA nanocages. Nanomedicine Nanotechnology, Biol. Med. 14, 1181–1190 (2018). 10.1016/j.nano.2018.02.002.

## RPC 05

### Mambalgin-2 selectively inhibits proliferation of ASIC1a-expressing cancer cells

#### Maxim Bychkov^1^, Mikhail Shulepko^1^, Natalia Vasylieva^2^, Olga Shlepova^1,3^, Anastasia Sudarikova^2^, Ekaterina Lyukmanova^1,3^

##### ^1^Shemyakin-Ovchinnikov Institute of Bioorganic Chemistry RAS, Moscow, Russia; ^2^Institute of Cytology RAS, St-Petersburg, Russia; ^3^Moscow Institute of Physics and Technology, Dolgoprudny, Moscow Region, Russia

ASICs and other members of the ENaC/Deg receptor family form the chimeric channels which are responsible for constitutive cation current in high-grade glioblastomas cells. Inhibition of these channels by the antagonists amiloride or spider toxin PcTx1 leads to decrease of the glioblastoma cell growth and invasion in vitro and in vivo. Previously, two peptides called mambalgin-1 and mambalgin-2 have been isolated from venom of Dendroaspis polylepis. It was shown that mambalgins, belonging to the family of three-finger toxins, are potent inhibitors of ASIC1a^1^. Recently, the authors have developed the high-efficient recombinant system for bacterial production of mambalgin-2. Here, the authors showed for the first time that recombinant mambalgin-2 reduced the growth of U251 MG and A172 glioma cells and A549 lung carcinoma cells, expressing the ASIC1a subunit, with EC50 ~0.9 ± 0.01 nM, ~10 ± 1.6 nM, and ~ 8 ± 0.2 nM, respectively. Mutant variants of mambalgin-2 with L32A and L34A substitutions, which abolish affinity towards ASIC1a channels, did not inhibit proliferation of U251 MG, A172, and A549 cells. Effect of mambalgin-2 on the glioma cell growth was comparable with the effect of amiloride. By electrophysiology the authors showed that mambalgin-2 inhibited amiloride-sensitive constitutive cation current through the cell membrane of U251 MG glioma cells. From the other hand, mambalgin-2 did not inhibit the growth of normal oral keratinocytes (Het-1A line) and epidermal carcinoma A431 cells which do not express ASIC1a subunit. These data point on ASIC1a as a selective marker in cancer cells for usage of mambalgin-2 like drugs for anticancer therapy. Our data revealed new functional properties of mambalgin-2 and point on the toxin as a useful hit in development of new antitumor drugs.

Disclosure: The study was supported by the Russian Foundation of Basic Research project № 18-34-00497 and by the President of Russian Federation № 4316.2018.4).

References

1. Bychkov, M. L. et al. Three-finger toxin Mambalgin-2 from Dendroaspis polylepis venom reduces the growth of glioblastoma cells by inhibition of ASIC1a channels. Toxicon 159, S24 (2019). 10.1016/j.toxicon.2018.11.398.

## RPC 06

### Oncolytic viruses for cancer treatment

#### Petr Chumakov^1,2^

##### ^1^V.A. Engelhardt Institute of Molecular Biology, Moscow, Russia; ^2^M.P. Chumakov Federal Scientific Center for Research and Development of Immune Biology Products, Moscow, Russia

The efficacy of systemic cancer therapy remains unacceptably low. The basis of modern systemic antitumor therapy is still chemotherapy and targeted drugs, which can prolong the lives of patients but do not significantly affect their five-year survival. In recent years, considerable attention is being attracted to cancer immunotherapy, which aims at stimulating the natural mechanisms of the destruction of tumor cells. Oncolytic viruses belong to a similar class of therapeutics. Viruses can not only selectively destroy tumor cells, but also significantly stimulate the mechanisms of antitumor immunity, overcome the suppressive effect of the tumor microenvironment. The great advantage of oncolytic viruses is their ability to destroy cancer stem cells, preventing the development of relapses of the disease. However, the disadvantage of oncolytic viral therapy is in the high degree of selectivity of individual tumors to the therapeutic effect of a particular strain of the oncolytic virus. When using a single viral preparation, only a small proportion of patients show a positive response. Moreover, tumors that are resistant to one viral strain may be highly sensitive to another viral strain. The authors are developing panels of oncolytic viruses with differences in the spectra of responses by individual tumors. From these panels, one can select personalized therapeutics for each patient. The authors are also developing diagnostic tests that can predict the sensitivity of a patient's tumor to individual viral strains from the therapeutic panel. The third area is the development of vehicles for delivering viruses to distant tumors and their metastases. Examples of the therapeutic effects of oncolytic viruses will be demonstrated, as well as safety data for oncolytic virus therapy.

## RPC 07

### Methyltransferase Set7/9 as a biomarker and potential target for therapy of lung and breast carcinomas

#### Alexandra A Daks^1^, Viktoria A Mamontova^1^, Alexey V Petukhov^1^, Oleg Y Shuvalov^1^, Olga A Fedorova^1^, Nickolai A Barlev^1^

##### ^1^Institute of Cytology RAS, St.-Petersburg, Russia

Methyltransferase Set7/9, a product of SETD7 gene, was firstly described as an enzyme that performs methylation of histone H3 on lysine 4 (H3K4). Set7/9 has a capability to methylate more than 30 non-histone proteins, which participate in differentiation, regulation of the gene expression and stress response to DNA damage. The latter ones include tumour suppressor genes such as RB, p65, Foxo3, DNMT1 and p53. Interestingly, Set7/9 methyltransferase can downregulate p53 activity and therefore modulate cell sensitivity to genotoxic agents. The authors hypothesize that clinically observed non-uniformity of lung cancer response to chemotherapy may depend on the status of Set7/9. With the help of CRISPR/Cas9 genomic editing system, the authors created the set of human lung cancer cell lines with the altered Set7/9 expression. Using the obtained cellular models and patients-derived cell lines, the authors showed that knockout of Set7/9 increases the sensitivity of lung cancer cells to genotoxic drugs doxorubicin and cisplatin, and also leads to the increase in the level of apoptosis in cells treated with cisplatin and etoposide. The authors have demonstrated that a specific inhibitor of Set7/9 methyltransferase activity, (R)-PFI-2, has a similar effect increasing their sensitivity to genotoxic drugs. The authors assume that the identified properties of Set7/9 are provided by its methyltransferase activity. Using bioinformatics analysis, the authors showed that increased Set7/9 expression is associated with shortened lifespan of patients with not only non-small cell lung cancer, but also those with breast cancer. In addition, the authors found that elevated level of Set7/9 is a characteristic of breast cancer of the HER2-positive subtype. Thus, the authors demonstrated the potential versatility of this methyltransferase as a tumor biomarker and a chemotherapy target.

Disclosure: This work was supported by Russian Science Foundation grant No 19-75-10059 and the Russian Federation Government grant for leading scientists No 14.W03.31.0029.

## RPC 08

### Chemotherapy as a modifier of tumor microenvironment

#### Oleg N Demidov^1,2,3^, Bogdan B Grigorash^1,2^, Elena Yu Kochetkova^2^, Burhan Uyanik^1^

##### ^1^Inserm UMR1231, UBFC, Dijon, France; ^2^Institute of Cytology RAS, St-Petersburg, Russia; ^3^Institute of Oncology, St-Petersburg, Russia

Chemotherapy remains the major way to treat cancer. It exploits cancer cells properties and induces tumor cell death and/or senescence that leads to elimination or shrinkage of the tumor. Normal cells including normal cells of tumor microenvironment are also affected by chemotherapy due to chemotherapy systemic action. Obviously, chemotherapy treatment induces DNA damage response not only in tumor cells but also in other organs and tissues. The authors study the role of DNA damage response genes in various immune cells of tumor microenvironment. Our results indicate that members of p53 signaling pathway actively involved in re-programing of anti-tumor immune response. The authors propose that effect of chemotherapy on tumor microenvironment should be considered in the development of new anti-cancer strategies. The new drugs targeting DNA damage response could be a new tool in modifying a tumor microenvironment and re-programming a tumor immunoresistance.

## RPC 09

### MDM2-Mediated Resistance of Lung Cancer Cell Lines to Genotoxic Drugs

#### Olga A Fedorova^1^, Oleg Y Shuvalov^1^, Alexandra A Daks^1^, Alexey V Petukhov^1^, Elena A Vasileva^1^, Nikolai A Barlev^1^

##### ^1^Institute of Cytology RAS, St-Petersburg, Russia

The proto-oncogene MDM2 is the main negative regulator of the p53 tumor suppressor protein. MDM2 interacts with at least 200 different proteins, participating in key signaling pathways of oncogenesis. The aim of this work is to investigate the mechanisms of MDM2-mediated resistance of cancer cells to genotoxic agents. To determine the spectrum of the MDM2-interacting proteins with or without treatment with genotoxic agents, the authors used the GST-pulldown followed by Mass Spectrometry to identify DNA repair factors interacting with MDM2. To confirmation of the most significant interactions, such as PARP1, Nibrin and Ku70 the authors carried out by co-immunoprecipitation. It is known that Nutlin binding to the amino terminal region of MDM2 inhibits the MDM2-p53 interaction, as well as with a number of other MDM2 interactions. The authors also study the effect of small-molecular inhibitor of p53-MDM2 interaction (Nutlin) on the spectrum of MDM2-binding proteins. The interaction of MDM2 with DNA repair factors can lead to their degradation or decrease in their activity, and respectively may affects on DNA repair. The functional significance of the identified interactions the authors determined by the identification of half-life of proteins (PARP1, Nibrin, Ku70). Also, the authors studied combined treatment of genotoxic drugs (doxorubicin, etoposide, cisplatin) and Nutlin in viability of different cell lines of lung cancer with different status of mdm2 and p53. The authors have shown that MDM2 enhances the resistance of the human lung adenocarcinoma (H1299) to doxorubicin. However, combined treatment with Nutlin and doxorubicin significantly increased the susceptibility of cells to genotoxic stress.

Disclosure: This study was funded by Russian Foundation for Basic Research grant № 18-315-20013.

## RPC 10

### The role of Hsp70 in transition of mesenchymal stromal cells towards cancer-associated fibroblasts

#### Anastasiia A Griukova^1^, Pavel I Deryabin^1^, Elena Y Komarova^1^, Irina V Guzhova^1^, Boris A Margulis^1^, Aleksandra V Borodkina^1^

##### ^1^Institute of Cytology RAS, St.-Petersburg, Russia

Homing is one of the most important features of mesenchymal stromal cells (MSCs) along with the capacity to self-renew and to differentiate into various cell lineages. In this context specific attention should be paid to Hsp70 protein, as it is crucial factor released by cancer cells and has potent protumorigenic properties. The present study aimed to investigate how cancer cells of different origin affect MSCs functioning and to reveal possible contribution of released Hsp70 in this interaction. Initially, the authors checked the effects of conditioned media (CM) collected from various cancer cell lines on the main ESCs properties. The authors revealed that CM from cancer cells despite of its origin caused significant alteration in ESCs morphology and reduction in proliferation rate. To test whether the observed morphological alterations might reflect ESCs differentiation towards cancer-associated fibroblasts (CAF) the authors estimated expression of the main CAF-marker genes. Indeed, ESCs cultured in CM obtained from cancer cells displayed enhanced expression of αSMA, FAP1, FSP1, and NG2, indicating acquisition of CAF phenotype. To investigate the role of released Hsp70 in the ESCs transition towards CAF, the authors further collected CM from wild-type, Hsp70-knocked-down and heat shock-stimulated cancer cells. The effects of Hsp70-depleted CM on ESCs proliferation and morphology were similar to those of wild-type CM, while the increased amount of Hsp70 partially reverted negative impact of cancer CM as indicated by the improved ESCs morphology and restored proliferation. However, the expression levels of CAF-maker genes in ESCs were unaffected by varying the content of Hsp70 in cancer cells CM. The authors can conclude that switch to CAF phenotype seems to be the preferable reaction of ESCs to the factors produced by various cancer cells. Also, the authors can assume that Hsp70 released by cancer cells probably is not the trigger of ESCs transition to CAF, but rather contributes to preservation of initial ESCs properties.

Disclosure: This study was funded by the Russian Science Foundation (#19-74-10038) and by the Russian Foundation for Basic Research (#18-29-09101-mk).

## RPC 11

### Inhibitors of molecular chaperones in anti-cancer therapeutics

#### Irina V Guzhova^1^, Vladimir F Lazarev^1^, Dmitry V Sverchinsky^1^, Alina D Nikotina^1^, Boris A Margulis^1^

##### ^1^Institute of Cytology RAS, St.-Petersburg, Russia

The success of monotherapy in practical oncology is limited and novel drug combinations are increasingly applied to enhance the efficacy of anti-cancer treatment. Since a tumor cell is guarded against proteotoxic surrounding and miscellaneous therapies by molecular chaperones the latter may be targeted to synergize the action of the traditional medicines. Using a few of assays the authors selected the substance able to inhibit Hsf1 activation in cancer cells and several compounds inhibiting chaperonic function of Hsp70; these compounds were found to act directly on the protein since they form a tight complex with Hsp70. Hsf1 inhibitor cardioglycoside CL-43 per se reduced tumor cell growth rate, colony formation and down-regulated the markers of epithelial-mesenchymal transition. Being applied in combination with a number of anti-cancer drugs CL-43 increased their anti-cancer capacity. Three inhibitors of Hsp70 function, two drug-like compounds and InCyt-11 peptide, were also found to enhance the pro-apoptotic activity of doxorubicin in cancer cells of several lineages. Importantly, this anti-cancer efficacy was also demonstrated in animal models of glioblastoma and melanoma. In conclusion the data show that chaperonic inhibitors may be employed in anti-cancer therapy as safe sensitizers of tumor cells to an action of currently employed drugs.

Disclosure: This work was supported by grant of Russian Scientific Foundation N 19-74-20161

## RPC 12

### Restoring impaired functions of mutant tumor suppressor p53 using synthetic modulators

#### Raniya R Khadiullina^1^, Regina M Mirgayazova^1^, Rimma N Mingaleeva^1^, Vitaliy V Chasov^1^, Matthias Baud^2^, Albert A Rizvanov^1^, Emil R Bulatov^1^

##### ^1^Kazan Federal University, Kazan, Russia; ^2^University of Southampton, United Kingdom

In almost half of all tumor cases the p53 is inactivated due to the mutations in DNA-binding domain sequence. One of the most common missence mutations is Y220C, which is observed in ~100,000 new diagnosed cancer cases annually worldwide. It results in the disturbance of the tertiary structure of the p53 DNA-binding domain, which in turn destabilizes the whole protein. Selective small molecule modulators of p53 (Y220C) mutant can be used to restore its impaired transcriptional functions. Here the authors investigated the interaction of 2nd generation derivatives of MB725 compound with recombinant p53 and p53-Y220C proteins. For that the authors used surface plasmon resonance to measure interaction Kd and differential scanning fluorimetry to estimate protein thermal stability in presence of the compounds. After that the authors used colorimetric MTS assay to evaluate cytotoxicity of the compounds using in cells with varying p53 status (wild type, knocknout, Y220C mutant). The compounds demonstrated a several fold increase in affinity to recombinant p53-Y220C compared to the previously described analogs, however induced thermal stability of p53-Y220C mutant increased modestly. The authors found that HUH7(p53-Y220C) and MCF7(p53-Y220C) were more sensitive to the treatment than MCF7(p53wt) and MCF7(p53-/-).

Disclosure: The study was supported by Russian Science Foundation grant #19-74-10022 to E.B., R.K. thanks Grant of the President of Russian Federation #MK-4253.2018.4, V.C. thanks Russian Fund for Basic Research #19-54-10005.

References

1. Bulatov, E. et al. Isatin-Schiff base-copper (II) complex induces cell death in p53-positive tumors. Cell Death Discov. 4, 103 (2018). 10.1038/s41420-018-0120-z.

2. Bulatov, E., Zagidullin, A., Valiullina, A., Sayarova, R. & Rizvanov, A. Small Molecule Modulators of RING-Type E3 Ligases: MDM and Cullin Families as Targets. Front. Pharmacol. 9, (2018). 10.3389/fphar.2018.00450.

3. Baud, M. G. J. et al. Aminobenzothiazole derivatives stabilize the thermolabile p53 cancer mutant Y220C and show anticancer activity in p53-Y220C cell lines. Eur. J. Med. Chem. 152, 101–114 (2018). 10.1016/j.ejmech.2018.04.035.

## RPC 13

### Autophagy impairment in Ki-Ras-mutated cancer cells through targeting the lysosomes results in cell death

#### Elena Yu Kochetkova^1^, Galina I Blinova^1^, Svetlana G Zubova^1^, Tatiana V Bykova^1^, Olga A Bystrova^1^, Marina G Martynova^1^, Valery A Pospelov^1^, Tatiana V Pospelova^1^

##### ^1^Institute of Cytology RAS, St.-Petersburg, Russia

Capability to restore viability through activation of cytoprotective autophagy is a factor that complicates antitumor therapy of cancers harboring mutated Ras. However, recent studies show that direct inhibition of autophagy does not succeed. Thus, to enhance cell death, an alternative strategy to inhibit the autophagic response is required. The present work aims on targeting lysosomes to impair autophagy in Ki-Ras-mutated human A549 non-small lung adenocarcinoma cells. We’ve compared the effects of acidification suppression (by inhibiting lysosomal v-ATPase with Bafilomycin A1) and direct suppression of autophagosome-lysosome fusion with Chloroquine on cellular viability. Both Bafilomycin A1 (Baf) and Chloroquine (CQ) were found to induce rough mitochondria damage in a short time, leading to decay of ATP levels, increase of Ulk1 Ser555 phosphorylation and autophagy induction. However, cells after Baf1 were unable to remove damaged mitochondria that led to massive cell death. In the same time, direct suppression of autophagosome-lysosome fusion by CQ didn’t suppress cellular viability as significantly as Baf, and Lysotracker revealed that lysosomal acidification is merely affected there unlike upon Baf treatment. We’ve compared effect of CQ in suppression of autophagosome-lysosome fusion with effect of cellular senescence, that is associated with low autophagosome-lysosome fusion intensity due to spatial compartmentalization of autophagosomes and lysosomes. It decreases intensity of autophagy, but cells remain viable. However, treatment of senescent cells with MEK/ERK kinase inhibitor leads to mitochondria damage that cannot be rescued due to attenuation of final stages of autophagy, linked with decrease of lysosome activity, and as the result cells undergo massive death. Thus, comparing cellular senescence and suppression of lysosomal acidification through inhibition of v-ATPase, it can be concluded that these both strategies allow to eliminate cells obtained from Ki-Ras-driven tumors.

## RPC 14

### Novel in vitro model of tumor repopulation

#### Lidia V Koludarova^1^, Dmitry V Sverchinsky^1^, Boris A Margulis^1^, Irina V Guzhova^1^

##### ^1^Institute of Cytology RAS, Saint-Petersburg, Russia

One of the most difficult problems of modern oncology is the tumor repopulation (TR) after therapy. Relapses appear after surgical removal of the tumor, as well as after applying radiation and chemotherapy. This phenomenon is observed in connection with the survival of a part of the population of cancer cells, in addition, their proliferation could be caused by factors released from dying and dead cells. Such factors are able to interact with surviving cancer cells and lead to the rapid recovery of the onconiche. The molecular mechanisms of TR have been shown to be associated with cell death processes triggered by therapeutic effects. In recent studies the most commonly used in vitro model of TR is based on co-culture of dying cells treated with anticancer agents as a source of proliferation inducing factors together with naïve cells. Despite of this model efficiency there are some drawbacks associated with limited opportunity to influence the signals releasing from dying cells. In our work the authors focused on development of in vitro TR model allowing to analyze the fractions bearing growth-stimulating factors. The authors obtained cell lysates, debris and conditioned media from cells treated with commonly used cell death inducers as rotenone, staurosporine and cisplatin. The conditioned media from cells treated with cisplatin had a stimulating growth effect on a small number of cancer cells, which was shown using the MTT assay, estimation of cell proliferative activity using the xCelligence technology and the colony formation test. The resulting model showed efficacy in several cell lines. In order to demonstrate the appropriacy of our model the authors generated A549 and DLD1 cells with knock-down of HSP70 chaperone, found to be involved in a cancer cell maintenance, and showed that the reduction of HSP70 either by knock-down in cells before drug application or directly in media by immunoprecipitation or by affinity chromatography diminishes the effect of repopulation in the present model. In conclusion, our model could provide better understanding in properties and roles of proteins involved in TR signaling.

Disclosure: This work was supported by Russian Science Foundation grant №19-74-20161.

## RPC 15

### Colorectal cancer cells expressing endogenous factors Oct4 and SOX2 as an adequate model for studying the stem component of a tumor

#### Sergey A Koshkin^1^, Alexander E Vinogradov^1^, Olga V Anatskaya^1^, Maria A Bistrakova, Elena N Tolkunova^1^

##### ^1^Institute of Cytology RAS, Saint-Petersburg, Russia

The initial stage of the study of tumor stem cells is the selection of an effective method of their enrichment. There is increasing evidence that the regulation of self-maintenance by activating the signal pathways of embryonic stem cells leads to the formation of the most aggressive tumor phenotype. To select and analyze colon cancer stem cells, the lentiviral reporter construction SORE6x^1^ was used for transduction of primary cell culture of adenocarcinoma. As a result, the authors obtained a line of cancer stem cells (CSC) of colon adenocarcinoma, selected on the basis of endogenous expression of transcription factors Oct4 and SOX2, binding with the promoter of Nanog^2^. The authors compared the global transcriptome of enriched CSC subpopulation with transcriptome of original cell line. Overall, our data identified 13206 protein-coding genes as differentially expressed at the traditional 2-fold threshold. Of these genes, 5495 genes were up-regulated and 7711 genes were down regulated. CSC demonstrated clear manifestations of the drug resistance and pluripotency and decreased proliferation. Main induced clusters were related to the regulation of pluripotency, development, male gametogenesis. Also, manifestation of stem cell properties were proven by the induction of Nodal signaling that promotes cell migration and EMT transition, and overexpression of the cluster of gametogenesis. In addition, the induced network contained several clusters related to drug metabolism and immunity. The down-regulated gene modules were related to cell growth, proliferation, cell cycle regulation and phase transition, aging, differentiation, chromatin remodeling, immune response and RNA metabolism. Unexpectedly, the down-regulated modules were presented by several pathways of apoptosis and numerous pathways of protein metabolism (both, protein synthesis and degradation), suggesting that drug resistance is promoted by depressed protein turnover. The authors have shown that enriched CSC subpopulation have increased chemo resistance, which considered as major characteristic of cancer stem cells and can be used as the model to study the potential ways to reverse this phenotype, by inhibition of the cell proliferation and self-renewal of the stem component of colon adenocarcinoma. They also can be used to search for additional prognostic factors and potential therapeutic targets for colon cancer treatment.

References

1. Tang, B. et al. A Flexible Reporter System for Direct Observation and Isolation of Cancer Stem Cells. Stem Cell Reports 4, 155–169 (2015). 10.1016/j.stemcr.2014.11.002.

2. Koshkin, S. et al. Primary cultures of human colon cancer as a model to study cancer stem cells. Tumor Biol. 37, 12833–12842 (2016). 10.1007/s13277-016-5214-8.

## RPC 16

### Development of new methods for treating glial tissue tumors

#### Galina Kosyakova^1,2^, Veronika Mikhailova^3^, Natalya V Mikhailova^1,2^

##### ^1^Research Institute of Pediatric Oncology and Hematology named after R.M.Gorbacheva, Moscow, Russia; ^2^First St. Petersburg State Medical University, Acad. I.P. Pavlova, Department of Biotechnology SIC PSPbGMU them. Acad. I.Pavlova, St.-Petersburg, Russia; ^3^Chemical-Pharmaceutical Academy. St.-Petersburg

Brain glioma occurs in 60% of tumors brain. To study and test a new laser removal of a tumor and high-intensity treatment focused ultrasound for the treatment of subcutaneous glioma the authors created three groups of animals.Two groups of rats were injected into the hemispheres of the brain. The third group of rats also glioma cells injected subcutaneously into the inner thigh of the hind leg. Within 1.5 -2months, a glioma developed in the hemisphere of the brain and in the subcutaneous tissue in the thigh, growing and becoming rounded and in some rats was noticeable above the surface within the brain Bone marrow was taken from all animals and peripheral smears were made.blood. One group of rats was a control, this group of tumor removal surgery brain surgeons did the usual surgical method. Second group animals removed glioma from the hemispheres of the brain with a laser apparatus. The third group of rats also received glioma cells subcutaneously in inner thigh of the hind leg. This group was used high-intensity focused ultrasound for the treatment of subcutaneous glioma. Germination of glioma of the brain in the brain membranes or bones of the skull were rarely observed only in exceptional cases. After repeated treatment with high-intensity focused ultrasound, glioma decreased in size under the skin. Brain glioma often had a rounded or spindle-shape, with size ranged from 2-3 mm in diameter in the brain and in the subcutaneous tissue on the inner thigh size sometimes reached the size of a chicken egg. In the study and testing of a new method of tumor removal by surgeons. At this was observed coagulation of the walls of the blood vessels of the brain. When studying and testing a new method for removing glioma laser apparatus, surgeons observed a lighter degree of wound healing. In practice, bleeding was not observed when the tumor was removed with a laser. Opposite in a group of rats with operable intervention without apparatus were small but persistent bleeding, there were small paresis of the limbs, sometimes cramps. After laser surgery in animals less commonly observed complications.

## RPC 17

### Exome analysis of vagal paragangliomas

#### Anna V Kudryavtseva^1^, Maria V Savvateeva^1^, Vladislav S Pavlov^1^, Dmitry V Kalinin^2^, Alexander L Golovyuk^2^, Maria S Fedorova^1^, Elena A Pudova^1^, Georgy S Razmakhaev^3^, Andrey A Poloznikov^3^, Anastasiya A Kobelyatskaya^1^, George S Krasnov^1^, Anastasiya V Snezhkina^1^

##### ^1^Engelhardt Institute of Molecular Biology, Russian Academy of Sciences, Moscow, Russia; ^2^Vishnevsky Institute of Surgery, Ministry of Health of the Russian Federation, Moscow, Russia; ^3^National Medical Research Radiological Center, Ministry of Health of the Russian Federation, Moscow, Russia

Vagal paraganglioma (VPGL) is a rare neuroendocrine tumor that arises from paraganglia associated with the vagus nerve. VPGL accounts for approximately 13% of all head and neck paragangliomas (HNPGLs). VPGL can occur both as familial and sporadic forms. Main susceptibility genes for VPGL are SDHx (SDHB, SDHC, and SDHD) encoding for subunits of succinate dehydrogenase (SDH) complex. VPGL is challenging in the diagnostics and treatment, and molecular genetic mechanisms involved in the tumor development have been poorly understood. The authors performed whole-exome sequencing of eight VPGLs using an Illumina technology. Exome sequencing and data analysis were carried out as described in our previous works^1,2^. To predict the pathogenicity of identified variants, the authors used the “pathogenicity score” that was calculated with population frequency, site conservation score, summary weighted score across prediction tools, and clinical significance annotation. Likely pathogenic variants in SDHx genes were revealed in six of eight VPGLs. In a patient, the authors found three likely pathogenic variants in the SDHB gene: one missense variant, NM_003000: c.A307G, p.M103V (chr1: 17355211, rs140178341), and two novel frameshift variants, NM_003000: c.308_309insTAAG, p.M103fs (chr1: 17355209) and NM_003000: c.304_305insATGAT, p.A102fs (chr1: 17355213). Splice acceptor variant, (chr1: 17350571, rs786201161), and stop-gain variant, NM_003000: c.C79T, p.R27X (chr1: 17371377, rs74315369), in the SDHB gene were also revealed in two other patients. Potentially pathogenic variant in the SDHD gene, NM_003002: c.A305G, p.H102R (chr11: 111959726, rs104894302), was found in two cases. Moreover, the authors determined likely pathogenic variants in SDHAF3 and SDHAF4, which encode for the assembly factors for SDH complex, as well as a variant in the IDH2 gene. No variants were identified in other potentially causative genes for HNPGLs (SDHA, VHL, TMEM127, RET, NF1, MAX, and others)^2,3^. However, likely pathogenic variants were identified in a number of tumor-associated genes, as well as novel ones. According to obtained results, in most cases, the development of VPGL seems to be driven by the disruption of SDH complex stability and function.

Disclosure: This work was supported by the Russian Science Foundation, grant no. 19-15-00419.

References

1. Kudryavtseva, A. V. et al. Mutational load in carotid body tumor. BMC Med. Genomics 12, 39 (2019). 10.1186/s12920-019-0483-x.

2. Snezhkina, A. V. et al. Exome analysis of carotid body tumor. BMC Med. Genomics 11, 17 (2018). 10.1186/s12920-018-0327-0.

3. Zhikrivetskaya, S. O. et al. Molecular markers of paragangliomas/pheochromocytomas. Oncotarget 8, (2017). 10.18632/oncotarget.15201.

## RPC 18

### The role of GAPDH in cancer cells resistance to hypoxia

#### Vladimir F Lazarev^1^, Mikhail.A. Mikeladze^1^, Elizaveta A Dutysheva^1^, Irina V Guzhova^1^, Boris A Margulis^1^

##### ^1^Institute of Cytology of RAS, St.-Petersburg, Russia

Tumor cells, including glioma cells, are in hypoxic conditions. With mild hypoxia, the proliferative activity of cancer cells increases, and with more severe hypoxia, protein denaturation and aggregation occurs, as a result of which apoptosis mechanisms are triggered. One of these proteins is glyceraldehyde-3-phosphate dehydrogenase (GAPDH), which is involved in glycolysis of the cell, which is especially important in conditions of hypoxia. The main goal of the work was to establish the relationship between the content of GAPDH in cancer cells and the prognosis of the development of a solid tumor under hypoxia, and also to propose a new approach for antitumor therapy using rat C6 glioma as an example. To accomplish our tasks, the authors used C6 line cells with increased and decreased level of gapdh expression. To obtain a rat model, the authors introduced C6 cells of various sublines into the Wistar rat striatum. As a therapeutic agent, the authors used the AEAC drug, which inhibits the antiaggregate activity of the cell's chaperone system through Hsp70 protein. The authors have demonstrated that as the gapdh expression level increases, the ability of cells to respond to hypoxia by activation of proliferation decreases. This is probably due to the fact that the appearance of the denatured form of GAPDH in cells initiated apoptosis, thus blocking proliferation. The AEAC compound significantly suppressed the interaction of Hsp70 and GAPDH, inhibiting hypoxia-induced cell proliferation. It is important to note that the effectiveness of antitumor therapy with AEAC was largely dependent on the level of gapdh expression. Finally, the authors tested AEAC on an animal model. It was found that rats with a brain tumor with a high GAPDH content lived longer than rats that were inoculated with a tumor with a low GAPDH content. Moreover, the effectiveness of therapy with AEAC was higher in the case of treatment of a tumor with increased expression of GAPDH. The data obtained by us allow us to outline the critical role of GAPDH in the development of a cancerous tumor and the mechanisms of regulation of carcinogenesis during hypoxia. The authors found that the resistance of glioma cells to hypoxia depends on the number of GAPDH in the cells and on its ability to aggregate.

Disclosure: This work was supported by grant from the Russian Scientific Foundation 19-74-20161.

## RPC 19

### Increased level of NF-kappaB affects proliferation and migration rates in lung cancer cells H1299

#### Ekaterina Lomert^1^, Ksenia Novitskaya^1^, Elizaveta Zhaivoron^1^, Dmitry Tentler^1^

##### ^1^Institute of Cytology RAS, St.-Petersburg, Russia

The evolutionarily conserved transcription factor NF-kappaB plays an important role in inflammation, immune response, and in almost all aspects of cellular activity. A number of studies showed prognostic significance of its expression level for outcome of patients with non-small cell lung cancer (NSCLC). However, impact of NF-kappaB on the proliferation and migration of cancer cells is still unexplored. Therefore the authors generated stable cell line H1299 with elevated level of RELA gene to model constitutive activation of NF-kappaB in NSCLC cells. Using immunostaining and RT-qPCR of target genes IKBA and ICAM1, the authors proved the nuclear localization and functional activity of the exogenous RelA/p65. The impact of the RelA/p65 subunit on the proliferation and motility of H1299 cells was analyzed using the xCELLigence RTCA DP system and CQ1 confocal quantitative image cytometer. Obtained growth curves showed that cells with increased level of RelA/p65 divided more slowly than the parent cells. Interestingly, the authors did not observe significant changes in cells cycle or apoptosis in H1299 cells with sustained RELA overexpression. At the same time the authors detected increased activity of senescence associated β-galactosidase which may be an indication of cellular senescence amplification in H1299/RelA line. The positive effects were shown on cell motility during wound healing assay and individual cell velocity measurements. Constant activation of RelA/p65 subunit led to accelerated wound healing compared to control cells. It is important to note that H1299 cells have a homozygous partial deletion of TP53 gene and lack expression of p53 protein. Therefore, all the observed changes in the proliferation and migration rates are p53-independent. As proof of this, sustained RELA overexpression in non-small cell lung cancer line H23 with mutant p53 leaded to increased cell proliferation rate. At the same time, cell lines with wild type p53 (A549 and H460) had the same phenotype as H1299 when RelA/p65 level was elevated. Thus, the effect of constant RelA/p65 activation on the proliferation of non-small cell lung cancer is cell line-specific and possibly depends on TP53 status.

Disclosure: This work was supported by the grant from the Russian Government Programme for the Recruitment of the leading scientists into the Russian Institutions of Higher Education 14.W03.31.0029

## RPC 20

### Small molecule modulators of mutant p53 demonstrate selective cytotoxicity toward p53-Y220C cell lines

#### Raniya R Khadiullina^1^, Regina M Mirgayazova^1^, Rimma N Mingaleeva^1^, Vitaliy V Chasov^1^, Matthias Baud^2^, Albert A Rizvanov^1^, Emil R Bulatov^1^

##### ^1^Kazan Federal University, Kazan, Russia; ^2^University of Southampton, United Kingdom

Transcription factor p53 plays a pivotal role in several critical cellular processes, including cell-cycle regulation, DNA repair and apoptosis. Missense-mutation Y220C is the ninth most frequent for p53 and is recorded globally in about 100,000 new cases annually of malignant tumors. Y220C mutation reduces thermal stability of the protein that causes disturbance of its tertiary structure and partial unfolding. A promising strategy of stabilizing the mutant p53 is based on using specific small molecule modulators that bind to narrow hydrophobic cavities formed as a result of mutation-induced changes in protein tertiary structure. The authors explored how 2nd generation derivatives of previously reported MB725 could restore biological activity of p53-Y220C mutant. As one of the biological models the authors use breast carcinoma MCF7(p53wt) and its two genetically modified variants, p53-/- and p53-Y220C, and also hepatocellular carcinoma HUH7(p53-Y220C). MCF7(p53-/-) was generated using CRISPR/Cas9 knockout. MCF7(p53-Y220C) was generated using lentiviral transduction of p53-Y220C into MCF7(p53-/-). Cytotoxicity of the compounds was assessed using colorimetric MTS assay. Cell proliferation and viability was monitored in real time using xCELLigence real time biosensor analysis. The compounds demonstrated substantial selectivity towards HUH7(p53-Y220C) and MCF7(p53-Y220C) cell lines compared to MCF7(p53wt) and MCF7(p53-/-).

Disclosure: The study was supported by Russian Science Foundation grant #19-74-10022 to E.B., Russian Fund for Basic Research #18-34-00702 to R.M., R.K. thanks Grant of the President of Russian Federation #MK-4253.2018.4.

References

1. Bulatov, E. et al. Isatin-Schiff base-copper (II) complex induces cell death in p53-positive tumors. Cell Death Discov. 4, 103 (2018). 10.1038/s41420-018-0120-z

2. Bulatov, E., Zagidullin, A., Valiullina, A., Sayarova, R. & Rizvanov, A. Small Molecule Modulators of RING-Type E3 Ligases: MDM and Cullin Families as Targets. Front. Pharmacol. 9, (2018). 10.3389/fphar.2018.00450

3. Baud, M. G. J. et al. Aminobenzothiazole derivatives stabilize the thermolabile p53 cancer mutant Y220C and show anticancer activity in p53-Y220C cell lines. Eur. J. Med. Chem. 152, 101–114 (2018). 10.1016/j.ejmech.2018.04.035

## RPC 21

### Development of magnetic particle-based theranostic agents with high specificity to cellular targets

#### Elizaveta N Mochalova^1,2^, Maria N. Yakovtseva^1^, S.P. Krechetov^1^, Vladimir R Cherkasov^1,2^, Maxim P Nikitin^1^

##### ^1^Moscow Institute of Physics and Technology (National Research University), Dolgoprudny, Russia; ^2^Prokhorov General Physics Institute of the Russian Academy of Sciences, Moscow, Russia

Magnetic particles (MPs) offer exciting opportunities in the field of targeted drug delivery due to their unique ability to be controlled with external magnetic fields^1–3^. The coupling of different recognition bioreceptors (for example, antibodies) to MPs provides their selective binding to certain cells, which allows creating effective agents for cancer therapy^4^. An important property of these agents is specificity, which ensures recognition and subsequent binding to predominantly malignant cells. In this work, the authors developed highly-specific MPs-based agents for cancer cell targeting. The authors synthesized a wide variety of MPs, which, together with commercial MPs, were functionalized with one of the most clinically successful monoclonal antibody Trastuzumab used to treat HER2/neu-positive breast cancer^5^. For developed agents, the authors investigated with high statistical significance the specificity of their interactions with cells using a number of magnetic and optical techniques, including imaging flow cytometry. For these purposes, the authors chose model cell lines with different levels of surface HER2/neu receptor expression. Additionally, the authors investigated two-stage targeting using biotin-labeled Trastuzumab and MPs conjugated with streptavidin. The authors demonstrated the influence of polymer coating on the non-specific interaction of the MPs with cells and chose the optimal polymers that reduce non-specific adhesion to the cells. Various approaches to particle functionalization were characterized – covalent immobilization and sorption, as well as the use of various recognition bioreceptors (Trastuzumab, streptavidin). Furthermore, imaging flow cytometry allowed a thorough investigation of interaction of MP-based agents with cells for different particle concentrations and cell lines, providing information about membrane targeting, clustering, and internalization. As a result, the authors demonstrate a comprehensive strategy for the development of agents capable of selectively binding to tumor cells overexpressing the HER2/neu receptor in the absence of binding to normal cells.

Disclosure: Different aspects of the research were partially supported by the Russian Fund for Basic Research grants No. 18-29-04065, 18-33-20252; Research Program 13 of Presidium of RAS; Grant of the President of the Russian Federation MK-5336.2018.3.

References

1. Tregubov, A. A., Nikitin, P. I. & Nikitin, M. P. Advanced Smart Nanomaterials with Integrated Logic-Gating and Biocomputing: Dawn of Theranostic Nanorobots. Chem. Rev. 118, 10294–10348 (2018). 10.1021/acs.chemrev.8b00198

2. Znoyko, S. L. et al. Ultrasensitive quantitative detection of small molecules with rapid lateral-flow assay based on high-affinity bifunctional ligand and magnetic nanolabels. Anal. Chim. Acta 1034, 161–167 (2018). 10.1016/j.aca.2018.07.012

3. Orlov, A. V., Pushkarev, A. V., Mochalova, E. N., Nikitin, P. I. & Nikitin, M. P. Development and label-free investigation of logic-gating biolayers for smart biosensing. Sensors Actuators B Chem. 257, 971–979 (2018). 971-979. 10.1016/j.snb.2017.11.025

4. Sokolov, I. L., Cherkasov, V. R., Tregubov, A. A., Buiucli, S. R. & Nikitin, M. P. Smart materials on the way to theranostic nanorobots: Molecular machines and nanomotors, advanced biosensors, and intelligent vehicles for drug delivery. Biochim. Biophys. Acta - Gen. Subj. 1861, 1530–1544 (2017). 10.1016/j.bbagen.2017.01.027

5. Lunin, A. V., Kolychev, E. L., Mochalova, E. N., Cherkasov, V. R. & Nikitin, M. P. Synthesis of highly-specific stable nanocrystalline goethite-like hydrous ferric oxide nanoparticles for biomedical applications by simple precipitation method. J. Colloid Interface Sci. 541, 143–149 (2019). 10.1016/j.jcis.2019.01.065

Russian Fund for Basic Research

## RPC 22

### Smyd2 inhibits p53 transcriptional activity by regulating its intracellular localization

#### Vasily M Muray^1^, Miran Rada^2^, Nikolai A. Barlev^1^

##### ^1^Institute of Cytology RAS, St.-Petersburg, Russia; ^2^University of Leicester, Leicester, UK

Lysine methyltransferase Smyd2 is currently under active investigation as an enzyme that regulates gene expression and participates in multiple signaling pathways. One of the most important targets of Smyd2 is the p53 protein, a central regulator of cell genome stability. The presence of Smyd2 protein decreases the concentration of promoter-bound p53, thus implementing a repressive effect on p53 target genes, and is potentially an oncogene and a target for cancer therapy. However, the mechanism of this repressive effect of Smyd2 on p53 has not yet been fully elucidated. Although the site of Smyd2-mediated methylation on the p53 protein at lysine 370 has already been discovered, the authors have shown that the methylation status of p53 at K370 does not affect binding of p53 to its target promoters. Thus, an assessment of relative luciferase activity and levels of mRNA expression of p53 target genes (namely, P21 and PUMA) did not reveal significant differences in the activity of the corresponding promoters in H1299 cell lines transfected with wild-type p53 or p53 K370/2R non-methylatable mutant. At the same time, there was a significant downregulation in the activity of target promoters upon overexpression of Smyd2 both in non-small lung carcinoma cells H1299 (p53 -/-) with ectopic p53wt and in H1299 cells expressing mutant p53K370/2R. Similar results were also shown by the colony formation assay. The authors hypothesized that this repressive mechanism is mediated via p53 protein retention in the cytoplasm by Smyd2, which hinders its transport to the nucleus. The results of immunocytochemistry and western blotting in H1299 cells and in MCF7 breast cancer cell line (p53wt) showed attenuated amounts of the p53 protein in the nucleus and increased p53 concentration in the cytoplasm in response to non-genotoxic stress (nutlin-3) and overexpression of Smyd2 when compared to control. These results indicate the presence of an indirect Smyd2-mediated action on the p53 protein and also suggest a new regulatory mechanism of p53 transcriptional activity.

Disclosure: This work was supported by Russian Science Foundation grant #19-45-02011.

## RPC 23

### Smart Nanomaterials as Advanced Sensors and Targeted Drug Delivery Agents

#### Maxim P Nikitin^1^

##### ^1^Moscow Institute of Physics and Technology (National University), Dolgoprudny, Russia

Theranostics, a fusion of diagnostics and therapy, promises to significantly improve the efficacy of medical treatment and to become the basis of personalized medicine. Ability to combine multiple functionalities in a single agent and react to external stimuli makes smart nanomaterials the perfect agents for this exciting new field of biomedicine^1,2^. Here, the authors will describe several novel approaches for fabrication of smart materials that can offer unconventional solutions to the challenging problems within various fields of theranostics. The first one is based on our recently proposed method of fabrication of biocomputing materials that can be designed to allow simultaneous analysis of concentration profiles of multiple molecular inputs (of virtually any nature) according to the rules of Boolean logic^3^. The authors have developed a multimodal magnetic and optical smart nanoagent, which is capable of sensing a variety of biomolecular markers and i) switching its affinity to a biomedical target as response to high concentrations of the marker, and ii) reporting the marker’s concentration in real-time via changing its optical properties^4^. Such unique combination of in situ biosensing capabilities with actuator functionality makes this approach attractive for development of novel tools for cell biology. Next, the authors developed novel superparamagnetic nanoparticles with a magnetite core in a porous metal organic framework shell^5^. These nanoparticles with stimuli-responsive shell can transport high amounts of drug payload and release it as result of biodegradation under the influence of phosphate ions (present in blood and other media of living systems. The authors demonstrated application of nanoparticles both as a multimodal and multifunctional agents. Further, the authors demonstrate novel magnetic reporters, namely, microagents based on magnetic vortex discs^6^ that allow: 1) their ultra-sensitive detection with magnetic particle quantification (MPQ) technique; 2) allow assessment of the localized viscosity (for in situ differentiation). Finally, the authors demonstrate 100-nm nanoagents that allow efficient treatment of pulmonary cancer metastases via enhanced drug delivery by RBC-hitchhiking^7^.

References

1. Tregubov, A. A., Nikitin, P. I. & Nikitin, M. P. Advanced Smart Nanomaterials with Integrated Logic-Gating and Biocomputing: Dawn of Theranostic Nanorobots. Chem. Rev. 118, 10294–10348 (2018). 10.1021/acs.chemrev.8b00198

2. Sokolov, I. L., Cherkasov, V. R., Tregubov, A. A., Buiucli, S. R. & Nikitin, M. P. Smart materials on the way to theranostic nanorobots: Molecular machines and nanomotors, advanced biosensors, and intelligent vehicles for drug delivery. Biochim. Biophys. Acta - Gen. Subj. 1861, 1530–1544 (2017). 10.1016/j.bbagen.2017.01.027

3. Nikitin, M. P., Shipunova, V. O., Deyev, S. M. & Nikitin, P. I. Biocomputing based on particle disassembly. Nat. Nanotechnol. 9, 716–722 (2014). 10.1038/nnano.2014.156

4. Shevchenko, K. G., Cherkasov, V. R., Tregubov, A. A., Nikitin, P. I. & Nikitin, M. P. Surface plasmon resonance as a tool for investigation of non-covalent nanoparticle interactions in heterogeneous self-assembly & disassembly systems. Biosens. Bioelectron. 88, 3–8 (2017). 10.1016/j.bios.2016.09.042

5. Tregubov, A. A. et al. Magnetic hybrid magnetite/metal organic framework nanoparticles: facile preparation, post-synthetic biofunctionalization and tracking in vivo with magnetic methods. J. Magn. Magn. Mater. 449, 590–596 (2018). 10.1016/j.jmmm.2017.10.070

6. Nikitin, M. P. et al. Ultrasensitive detection enabled by nonlinear magnetization of nanomagnetic labels. Nanoscale 10, 11642–11650 (2018). 10.1039/C8NR01511B

7. Zelepukin, I. V. et al. Nanoparticle-based drug delivery via RBC-hitchhiking for the inhibition of lung metastases growth. Nanoscale 11, 1636–1646 (2019). 10.1039/C8NR07730D

## RPC 24

### Regulation of p53 tumor suppressor by ZEB1 master regulator of epithelial-mesenchymal transition in breast cancer cells

#### Sergey E Parfenyev^1^, Nickolai A Barlev^1,2^

##### ^1^Institute of Cytology RAS, St.-Petersburg, Russia; ^2^Moscow Institute of Physics and Technology (National Research University), Dolgoprudny, Russia

The epithelial-mesenchymal transition (EMT) is a key mechanism determining the ability of cancer cells to form metastases. During this process, the expression of a number of genes changes leading to a disruption of the stable intercellular adhesion, which in most cases is associated with the suppression of CDH1 gene that codes for a transmembrane protein E-cadherin. Transcription factor ZEB1 serves as one of the main regulators EMT. ZEB1 also inhibits the expression of CDH1 gene. In turn, the tumor suppressor protein p53 inhibits ZEB1 through p53-dependent micro-RNAs, thereby preventing metastases. In the current work, the authors have used a model breast cancer cell system, which has an epithelial origin (MCF7) but can induce the expression of Zeb1 in response to doxycycline (Tet-ON). Using this system (MCF7-ZEB1), the authors found that induced overexpression of ZEB1 gene in leads to a decrease both in the mRNA and protein level of p53. However, a combination of ZEB1 induction and treatment with genotoxic drug doxorubicin gives opposite effect. The expression level of TP53 significantly increases compared to one in control and cells with induced ZEB1 without treatment with doxorubicin. A similar effect occurred with respect to p53 target genes: p21 and PUMA. Also, an increase in ZEB1 causes an increase of resistance of MCF7 cells to genotoxic drugs by stopping the cell cycle. This was confirmed by an increase in the p27 protein level, which is known as a cell cycle repressor. There was also a decrease in apoptosis in MCF7 cells with induced ZEB1 gene expression after the addition of doxorubicin. Moreover, the activation of ZEB1 shows an increase in the level of DNA-PK protein kinase that is a key component of non-homological DNA repair in particular NHEJ which leads to mutations. PRKDC (encodes DNA-PK) expression is known to correlate with poor prognosis in some types of tumors. Interestingly, ZEB1 restores VIM gene expression otherwise downregulated by addition of the doxorubicin and simultaneously inhibits CDH1 transcription promoting EMT which indicates a decrease in the therapeutic effect of the drug doxorubicin by ZEB1 acting.

Disclosure: This work was supported by RSF grant # 19-45-02011 and the Russian Government Programme for the Recruitment of the leading scientists into the Russian Institutions of Higher Education 14.W03.31.0029.

## RPC 25

### Proof of concept and prospects for the use of molecularly imprinted polymer nanoparticles («plastic antibodies») in cancer therapy

#### Alexey V Petukhov^1^, Alexandra A Daks^1^, Olga A Fedorova^1^, Oleg Y Shuvalov^1^, Nickolai Barlev^1,2^

##### ^1^Gene Expression Program, Institute of Cytology, St.-Petersburg, Russia; ^2^Moscow Institute of Technology and Physics, Dolgoprudny, Moscow Region, Russia

To date, monoclonal antibodies are widely used in antitumor therapy. The main mechanism of the therapeutic effect of monoclonal antibodies is antibody-dependent cellular cytotoxicity (ADCC). A monoclonal antibody binds to an antigen on the surface of a tumor cell by a variable region, while the constant region Fc remains free to interact with cells of the immune system. Natural killers expressing CD16A recognize the Fc region and eliminate tumors. Another mechanism of antibody therapy is Complement Dependent Cytotoxicity (CDC). Despite this, next-generation antibodies are combined with cytotastics, which in combination with chemotherapy gives the chances of achieving a complete remission necessary, for example, for allogeneic bone marrow transplantation. However, monoclonal antibodies have significant disadvantages such as immunogenicity, the need for combination with chemotherapy and high cost. Supermolecular polymer particles that the authors called molecularly imprinted polymer nanoparticles (nanoMIPs) are deprived of these drawbacks. Using triple-negative breast cancer cell lines, the authors proved the specificity of such plastic antibodies for delivering doxorubicin to tumor cells through recognition of the EGFR receptor. Anti-EGFR nanoMIPs were produced specific to SLNITSLGLRSLKEISDG peptide by solid-phase synthesis. In addition, nanoMIPs had cavities for binding to doxorubicin in one embodiment and a green fluorescent label in another. The authors incubated the MIPs in a solution of doxorubicin in water (final concentration 50 μM). Then the MIPs were washed with Amicon filters and tested. The concentration of MIP samples was 350 μg/ml. After that, the authors incubated nanoMIP at the concentration of 10 μg/ml with MDA-MB-468 and SKBR-3 cell lines in DMEM supplemented with 10% FBS. The evaluation of binding to MIPs was performed by flow cytometry and fluorescence microscopy. During incubation of 2 or 24 h there was a clear correlation between nanoMIPS binding and EGFR expression level on the cell lines detected by Western blotting. The authors hope that this approach will also be effective in relation to checkpoint inhibitor therapy, which will allow to fulfill highly effective targeted chemotherapy with low doses and a simultaneous immune response against the tumor leading to the eradication of the tumor clone.

Disclosure: This work was supported by grants Russian Fund for Basic Research#18-29-09144, Russian Science Foundation#18-75-10076.

## RPC 26

### The novel potential metastasis biomarkers in Zeb1 interactome after EMT induction in MCF7 breast carcinoma cells

#### Danila Y Pozdnyakov^1^, Sergey V Shabelnikov^1^, Alexey G Mittenberg^1^

##### ^1^Institute of Cytology, RAS, St.-Petersburg, Russia

Breast cancer is the most frequently diagnosed malignant neoplasm, as well as the second leading cause of death among women with cancer. The process of metastasis is of particular importance in carcinogenesis and most often cause of deaths. The epithelial-mesenchymal transition is one of the main pathways for the formation of secondary tumor nodules and a promising area for the search for therapeutic targets. The Zeb1 transcription factor, which plays an important role in carcinogenesis, is one of the main regulators of the epithelial-mesenchymal transition. Zeb1 activity is regulated by interacting with various proteins, among which potential targets were searched for therapeutic effects on the Zeb1 factor and metastasis. Based on the foregoing, the aim of the work was to search potential targets for influencing the metastasis of breast cancer in the Zeb1 interactome. To achieve the goal, it was necessary to induce an epithelial-mesenchymal transition in breast carcinoma cells and confirm it with both fluorescence microscopy and immunoblotting; immunoprecipitate the Zeb1 interactome, followed by mass spectrometric identification of its constituent proteins; analyze the Zeb1 interactome-proteins and evaluate their possible role in the epithelial-mesenchymal transition. Zeb1 interactome was isolated with immunoprecipitation from breast carcinoma cells at different stages of the epithelial-mesenchymal transition and the proteins were identified by LC-MALDI TOF/TOF mass spectrometry. According to the results obtained, the following conclusions were made: 1. Using immunoblotting, changes in the specific protein markers expression, vimentin (mesenchymal) and E-cadherin (epithelial), were confirmed after EMT induction in MCF7/Zeb1 cells. 2. The method of tandem time-of-flight mass spectrometry demonstrated significant differences in protein composition of Zeb1 interactome in the early (24 h) and late (72 h) EMT stages. 3. Among the Zeb1 interactome proteins a number of promising targets for the development of new anti-cancer therapeutic approaches (ADAR, DDX17, CTBP2, SRussian Science Foundation3, MPG, TDP-43) were detected.

Disclosure: The work was supported by the Russian Foundation for Basic Research (project 18-29-09073-MK (A.G.M.)).

## RPC 27

### Differentially expressed microRNAs associated with lymph node dissemination in locally advanced prostate cancer

#### Elena A Pudova^1^, Anastasiya A Kobelyatskaya^1^, Kirill M Nyushko^2^, Maria VSavvateeva^1^, Nataliya V Melnikova^1^, Andrey D Kaprin^2^, Boris Y Alekseev^2^, Anastasiya V Snezhkina^1^, George S Krasnov^1^, Anna V Kudryavtseva^1^

##### ^1^Engelhardt Institute of Molecular Biology RAS, Moscow, Russia; ^2^National Medical Research Radiological Center, Ministry of Health of the Russian Federation, Moscow, Russia

Prostate cancer (PCa) is malignancy that rank third in male mortality in developed countries. Existing biomarkers (Gleason score and clinical pathological characteristics) have insufficient predictive value. The aim of this study is to find out microRNAs with differential expression between lymph-node negative (N0) and positive (N1) locally advanced prostate cancer (LAPC) in Russian patients. The authors performed miRNA-Seq for 47 tumor samples taken from LAPC patients (34 N0 and 13 N1) that underwent surgical treatment and did not receive neoadjuvant therapy. Total RNA isolated by MagNA Pure Compact RNA Isolation Kit (Roche, Switzerland). Libraries were prepared from RNA with RIN no less than 7.0 by NEBNext® Small RNA Library Prep Set for Illumina (New England Biolabs, USA). miRNA-Seq was performed on NextSeq500 System (platform Illumina) at the EIMB RAS “Genome” Center [http://www.eimb.ru/rus/ckp/ccu_genome_c.php]. To process miRNA-Seq data, the authors used miRge 2.0 pipeline. For differential expression analysis of miRNA counts the authors used edgeR package. The authors compared miRNA expression profiles between N0 and N1 groups and identified at least 17 differentially expressed miRNAs, which have passed thresholds: FDR < 0.05, both Mann-Whitney and quasi-likelihood tests; no less than 2-fold expression level changes between N0 and N1. Interestingly, the authors did not observe such strong association of the expression of these microRNAs with N0-N1 status according to TCGA data. According to our data, among them, 8 miRNAs were upregulated in N1 group: hsa-miR-20a-5p, miR-106a-5p/17-5p, miR-93-5p, miR-15b-5p, miR-203a-3p, miR-16-2-3p, miR-452-5p, miR-454-3p (3—8-fold upregulation). Also, the authors identified 9 downregulated miRNAs in N1 samples: hsa-miR-6842-3p, miR-370-3p, miR-671-3p, miR-589-5p, miR-7706, miR-941, miR-365a-5p, miR-184, miR-6510-3p (3—6-fold decreased expression level). Thus, these microRNAs can represent potential markers of either prognosis and/or lymphatic dissemination of locally advanced prostate cancer.

Disclosure: This work was funded by the Russian Science Foundation, grant 18-75-10127.

## RPC 28

### Effect of structural features of MDM2 inhibitors on expression of p53 target genes

#### Angelina A Romanova^1^, Mariya Yu Lvova

##### ^1^Laboratory of Molecular Pharmacology, St.-Petersburg State Institute of Technology (Technical University), St.-Petersburg, Russia

Disturbances in the activity of key signaling proteins that control various aspects of cell functioning are often the cause of the development of complex diseases such as metabolic syndrome and malignant neoplasms^1,2^. The stimulation of the function of the pro-apoptotic p53 protein by inhibiting its binding to the E3-ubiquitin ligase MDM2, which results in the inhibition of the transcriptional activity of p53 and its ubiquitin-dependent degradation, is one of approaches to compensate hyperproliferative phenomena. Pharmacological suppression of the p53-MDM2 interaction leads to the stabilization of p53 and stimulation of its antitumor activity, including by triggering transcription of target genes^3^. However, in the case of each certain small molecule p53 reactivator, the question “what cellular effect will dominate—apoptosis or cell cycle arrest” remains open. In the latter case, the risk of secondary tumor development from surviving cells, which are often not sensitive to a wide range of drugs, increases significantly. Thus, the determination of the predominant cellular mechanism triggered by a particular p53 reactivator is a necessary step in the development of the drug. The aim of the work was to study the effect of MDM2 inhibitors belonging to different chemical classes on the transcriptional activity of the p53 protein. For this purpose, the authors developed a procedure and conditions for treating HCT-116 cells with biologically active substances: cis-imidazoline, indolinone, and iso-indolinone derivatives^4–6^. Expression of p53 target genes responsible for cell cycle arrest (p21, 14-3-3) and apoptosis (PUMA, NOXA) were quantified by real-time reverse transcription polymerase chain reaction (RT-PCR) after 24 h of the cultivation with the studied substances. As a result, it was shown that representatives of different chemical classes preferably activate different targets: significant differences were observed in relative expression of PUMA/NOXA/14-3-3/p21, which can lead to fundamentally different variants of the cell fate. Thus, it is expected that the analysis of transcription mechanisms activated by one or another MDM2 inhibitor will allow more efficient development of drugs and screening out candidates with a high risk of chemoresistance.

Disclosure: This work was supported by the Russian Science Foundation (project no. 19-73-10150).

References

1. Zhigacheva, I. V. & Burlakova, E. B. Plant Growth and Development Regulators and their Effect on the Functional State of Mitochondria. in Chemistry and Technology of Plant Substances 243–278 (Apple Academic Press, 2017). 10.1201/9781315207469-12.

2. Novikova, D. S., Garabadzhiu, A. V., Melino, G., Barlev, N. A. & Tribulovich, V. G. Small-molecule activators of AMP-activated protein kinase as modulators of energy metabolism. Russ. Chem. Bull. 64, 1497–1517 (2015). 10.1007/s11172-015-1036-x.

3. Krasavin, M. et al. Design, in silico prioritization and biological profiling of apoptosis-inducing lactams amenable by the Castagnoli-Cushman reaction. Bioorg. Med. Chem. 26, 2651–2673 (2018). 10.1016/j.bmc.2018.04.036.

4. Novikova, D. S., Grigoreva, T. A., Zolotarev, A. A., Garabadzhiu, A. V. & Tribulovich, V. G. Advanced palladium free approach to the synthesis of substituted alkene oxindoles via aluminum-promoted Knoevenagel reaction. RSC Adv. 8, 34543–34551 (2018). 10.1039/C8RA07576J;

5. Orlova, D. D., Novikova, D. S., Garabadzhiu, A. V. & Tribulovich, V. G. A Study on Hydrolytic Stability of Isatin N-Mannich Bases. Russ. J. Gen. Chem. 88, 48–56 (2018). 10.1134/S1070363218010085;

6. Grigoreva, T. A., Novikova, D. S., Gureev, M. A., Garabadzhiu, A. V. & Tribulovich, V. G. Amino acids as chiral derivatizing agents for antiproliferative substituted N-benzyl isoindolinones. Chirality 30, 785–797 (2018). 10.1002/chir.22854.

## RPC 29

### MIL-100 coated nanoparticles as a potential tool for in vitro delivery of therapeutic agents

#### Alina Ringaci^1^, Konstantin G Shevchenko^1,2^, Vladimir R. Cherkasov^1,2^, Evgenii L. Kolychev^1,2^

##### ^1^Moscow Institute of Physics and Technology, Moscow, Russia; ^2^Prokhorov General Physics Institute, Moscow, Russia

One of the cancer hallmarks is complexity, variety and adaptivity of its underlying mechanisms. Combination of gene and chemotherapy with synergistic reinforcement of the effect from both strategies is considered to be the perspective strategy for drug development. In this respect nanotechnology offers broad opportunities for creating smart systems for research, diagnostics and therapy^1–3^. Metal-organic frameworks (MOFs), a crystal structuresconstructed from metal ions that are linked by organic molecules, have been steadily emerging through the last decade as a promising agent for biomedical applications^4^. Due to their porous structure and low biotoxicity they can effectively adsorb, deliver and release therapeutic compounds where and when required. However, until now MOF biomedical applications were limited to delivery of molecules that were capable of being adsorbed into the pores, whereas their vast exterior surface was not used as a payload capacity. Here the authors propose a concept of a MOF-based vector for combined in vitro delivery of several therapeutic agents. To check this concept, the authors developed a MIL-100 shell coated superparamagnetic nanoparticles with a model small molecule adsorbed inside. Next, the authors checked the efficacy of such vector for in vitro delivery in several cell lines. For the small molecule the transfection rate correlated with the rate of cells that accumulated nanoparticles and proved the low toxicity of the MOF. The vector can be used as a tool for cancer research and therapy.

Disclosure: This study was funded by Russian Fund for Basic Research grant#19-33-70075 and Russian Science Foundation grant# 18-73-10102.

References

1. Tregubov, A. A., Nikitin, P. I. & Nikitin, M. P. Advanced Smart Nanomaterials with Integrated Logic-Gating and Biocomputing: Dawn of Theranostic Nanorobots. Chem. Rev. 118, 10294–10348 (2018). 10.1021/acs.chemrev.8b00198

2. Shevchenko, K. G., Cherkasov, V. R., Tregubov, A. A., Nikitin, P. I. & Nikitin, M. P. Surface plasmon resonance as a tool for investigation of non-covalent nanoparticle interactions in heterogeneous self-assembly & disassembly systems. Biosens. Bioelectron. 88, 3–8 (2017). 10.1016/j.bios.2016.09.042

3. Shevchenko, K. G., Cherkasov, V. R., Nikitina, I. L., Babenyshev, A. V. & Nikitin, M. P. Smart multifunctional nanoagents for in situ monitoring of small molecules with a switchable affinity towards biomedical targets. Appl. Nanosci. 8, 195–203 (2018). 10.1007/s13204-018-0659-2

4. Tregubov, A. A. et al. Magnetic hybrid magnetite/metal organic framework nanoparticles: facile preparation, post-synthetic biofunctionalization and tracking in vivo with magnetic methods. J. Magn. Magn. Mater. 449, 590–596 (2018). 10.1016/j.jmmm.2017.10.070

## RPC 30

### Development of the enzyme-responsive nanoparticle-based system for targeted drug delivery

#### Konstantin G Shevchenko^1,2^, Alina Ringaci^1^, Andrei V. Babenyshev^1,2^, Maxim P Nikitin^1^

##### ^1^Moscow Institute of Physics and Technology (National Research University), Dolgoprudny, Russia; ^2^Prokhorov General Physics Institute of the Russian Academy of Sciences, Moscow, Russia

In the last decade advances in deciphering the molecular mechanisms of tumour development has stimulated development of the new generation of drugs that preferably target cancer cells. The next step within this paradigm would be generation of the multifunctional agents, that are capable of simultaneous analysis of its environment and specific release of the drug only in the presence of tumour biomarkers. Nanoparticles present one of the most effective platforms to carry out such task due to their unique chemical and biological properties^1,2^. Besides, their surface can be easily modified with antibodies, receptors, enzymes or other biomolecules, that can recognize and bind specific target^3,4^. For instance, MMP7 and MMP9 proteases could be utilized as a specific molecule for tumour-environment recognition. Here the authors show the new smart enzyme-responsive nanoparticle-based system as a potential tool for biomedical applications. The authors assembled a multilayer nanostructure that utilized magnetic nanoparticle as the core with gold nanoparticles immobilized on its surface. As the interface for enzyme recognition and a link between core and surface particles the authors used proteins, such as BSA, that were specifically broken down by model peptidases. Upon addition of the enzyme, the system changed its configuration, which was supported by SEM and SPR shift^5^ analysis, and the surface of the core became exposed to the environment. So, the proposed system is an attractive platform for development of enzyme based targeted drug delivery and biosensing.

Disclosure: Different parts of the reported study were supported by Russian Fund for Basic Research, project number 18-03-01252, Presidium of RAS, the Basic Research Program I.7, and President Programme to Support Young Scientists grant N 075-02-2018-099.

References

1. Nikitin, M. P., Shipunova, V. O., Deyev, S. M. & Nikitin, P. I. Biocomputing based on particle disassembly. Nat. Nanotechnol. 9, 716–722 (2014). 10.1038/nnano.2014.156

2. Shevchenko, K. G., Cherkasov, V. R., Nikitina, I. L., Babenyshev, A. V. & Nikitin, M. P. Smart multifunctional nanoagents for in situ monitoring of small molecules with a switchable affinity towards biomedical targets. Appl. Nanosci. 8, 195–203 (2018). 10.1007/s13204-018-0659-2

3. Tregubov, A. A., Nikitin, P. I. & Nikitin, M. P. Advanced Smart Nanomaterials with Integrated Logic-Gating and Biocomputing: Dawn of Theranostic Nanorobots. Chem. Rev. 118, 10294–10348 (2018). 10.1021/acs.chemrev.8b00198

4. Sokolov, I. L., Cherkasov, V. R., Tregubov, A. A., Buiucli, S. R. & Nikitin, M. P. Smart materials on the way to theranostic nanorobots: Molecular machines and nanomotors, advanced biosensors, and intelligent vehicles for drug delivery. Biochim. Biophys. Acta - Gen. Subj. 1861, 1530–1544 (2017). 10.1016/j.bbagen.2017.01.027

5. Shevchenko, K. G., Cherkasov, V. R., Tregubov, A. A., Nikitin, P. I. & Nikitin, M. P. Surface plasmon resonance as a tool for investigation of non-covalent nanoparticle interactions in heterogeneous self-assembly & disassembly systems. Biosens. Bioelectron. 88, 3–8 (2017). 10.1016/j.bios.2016.09.042

## RPC 31

### Membrane-bound heat shock protein Hsp70 as a theranostic target for cancer therapy

#### Maxim A Shevtsov^1,2,3,4^, Boris P Nikolaev^5^, Yaroslav Yu Marchenko^5^, Ludmila Yu Yakovleva^5^, Gabriele Multhoff^1^, Irina V Guzhova^2^, Boris A Margulis^2^

##### ^1^Center for Translational Cancer Research Technische Universität München (TranslaTUM), Klinikum Rechts der Isar, Munich, Germany; ^2^Institute of Cytology RAS, St-Petersburg, Russia; ^3^First Pavlov State Medical University of St-Petersburg, St.-Petersburg, Russia; ^4^Almazov National Medical Research Centre, Russian Polenov Neurosurgical Institute, St.-Petersburg, Russia; ^5^Research Institute of Highly Pure Biopreparations, St.-Petersburg, Russia

Heat shock protein 70 (Hsp70), also termed HSPA1A, is overexpressed in a large variety of different tumor types^1^. Apart from its intracellular localization, a tumor-specific Hsp70 membrane expression was discovered. For targeting mHsp70 on tumor cells various nanocarriers were synthesized including superparamagnetic iron oxide nanoparticles (SPIONs), gold nanoparticles (AuNPs) and CdSe quantum dots (QDs)^2,3^. To provide the tumor-specific localization of the nanoparticles mHsp70 targeting bioligands (i.e., anti-Hsp70 monoclonal antibody cmHsp70.1, serine protease granzyme B, tumor-penetrating peptide (TPP)) were employed for the functionalization of the particles surface^2–4^. Tumor selectivity and cytotoxicity of the obtained conjugates was assessed in series of in vitro experiments using murine and human tumor cell lines by flow cytometry analysis, confocal and electron microscopies. In vivo targeting potential of nanoparticles was assessed in the models of orthotopic human U87 glioblastoma and orthotopic human small cell lung carcinoma H1339 in NMRI nu/nu mice, orthotopic B16 melanoma in C57/Bl6 mice, orthotopic rat C6 glioblastoma in Wistar rats. Additionally, combinatorial treatment regimens employing stereotactic radiotherapy, magnetic targeting and/or immune checkpoint inhibitors in combination with mHsp70-targeting nanoparticles were assessed in the clinically relevant animal models. In vitro experiments demonstrated the selectivity of mHsp70 targeting conjugates in different tumor cell types. Unique physico-chemical properties of various synthesized nanoparticles provided the broad spectrum of diagnostic and therapeutic applications in translational oncology. In vivo studies showed tumor-specific accumulation of the functionalized nanoparticles that resulted in the significant contrast enhancement of the tumors (based on MRI for the SPIONs and epifluorescence microscopy for CdSe QDs). Application of the serine protease granzyme B (GrB), that has a pro-apototic activity, for the decorating of the nanoparticles surface induced specific tumor cell apoptosis. Combinatorial regimens using stereotactic radiotherapy and/or magnetic targeting further enhanced the therapeutic efficacy of GrB coated SPIONs in different tumor mouse models. Further addition of the immune checkpoint inhibitors (anti-CTLA-4 and anti-PD-1 monoclonal antibodies) increased the overall survival of animals. Membrane-bound Hsp70 could be used as a target for novel nanoparticle-based approaches for the treatment of cancer.

References

1. Shevtsov, M., Huile, G. & Multhoff, G. Membrane heat shock protein 70: a theranostic target for cancer therapy. Philos. Trans. R. Soc. B Biol. Sci. 373, 20160526 (2018). 10.1098/rstb.2016.0526

2. Shevtsov, M. A. et al. Ionizing radiation improves glioma-specific targeting of superparamagnetic iron oxide nanoparticles conjugated with cmHsp70.1 monoclonal antibodies (SPION–cmHsp70.1). Nanoscale 7, 20652–20664 (2015). 10.1039/c5nr06521f

3. Shevtsov, M. et al. Granzyme B Functionalized Nanoparticles Targeting Membrane Hsp70‐Positive Tumors for Multimodal Cancer Theranostics. Small 15, 1900205 (2019). 10.1002/smll.201900205

4. Stangl, S. et al. Preclinical Evaluation of the Hsp70 Peptide Tracer TPP-PEG 24 -DFO[89 Zr] for Tumor-Specific PET/CT Imaging. Cancer Res. 78, 6268–6281 (2018). 10.1158/0008-5472.CAN-18-0707

## RPC 32

### MDM2 ubiquitin-ligase down-regulates energy metabolism of human tumor cell models

#### Oleg Y Shuvalov^1^, Alexey V Petukhov^1,2^, Olga A Fedorova^1^, Alexandra A Daks^1^, Anastasiya V Snezhkina^3^, Nickolai Barlev^1^

##### ^1^Institute of cytology RAS, St-Petersburg, Russia; ^2^Almazov national medical research centre, St-Petersburg, Russia; ^3^Engelhardt Institute of Molecular Biology, Moscow, Russia

MDM2 is an E3 ubiquitin ligase that is the main negative regulator of p53 tumor supressor. MDM2 continuously ubiquitinates p53 targeting it for proteasomal degradation. However, cancer cells frequently use this mechanism to protect from p53-dependent cell death. Besides negative regulation of p53, MDM2 possess other both oncogenic and oncosupressive properties. As example, it targets for degradation catalytic subunit of telomerase (hTERT), epithelial-to-mesenchymal transition factors Snail and Slug, oncosupressor Rb, etc. To identify new interactants of MDM2, we have carried out GST pull-down of MCF7 and U2OS cell extracts using GST-MDM2 as bait followed by liquid chromatography coupled with mass-spectrometry (LC-MS/MS). We have identified 232 proteins associated with MDM2. Among them there were key enzymes of glycolysis – PKM, ALDOA and LDHA which are frequently deregulated in various malignancies. Using cell lines with overexpression and knockdown of MDM2, we have studied the influence of MDM2 on protein level of PKM, ALDOA and LDHA, their ubiquitination and stability. We have also studied the influence of MDM2 on energy metabolism including glycolysis and respiration. The MDM2 status significantly altered the susceptibility of cancer cell models to chemotherapeutic inhibitors of glycolysis and respiration. Up-regulated glycolysis is considered now as one of the “Hallmarks of cancer” making its enzymes as promising targets for antitumor drugs. This point emphasizes the importance of MDM2 for tumor-associated metabolism and warrants further investigations.

Disclosure: This work was supported by RSF grant # 19-45-02011 and the Russian Government Programme for the Recruitment of the leading scientists into the Russian Institutions of Higher Education 14.W03.31.0029.

## RPC 33

### Tissue Transglutaminase as an emerging target therapy in cancer and the microbial enzyme as the new tool for the development of Antibody drug Conjugate

#### Eva Sivado^1^, Meddy El Alaoui^1^, Mike R Dyson^3^, Sandrine Valsesia-Wittmann^2^, Said El Alaoui*^1^

##### ^1^Covalab, Villeurbanne, France; ^2^Centre Léon Bérard, Immunotherapy lab-P3I, Lyon, France; ^3^IONTAS Ltd, Pampisford, Cambridge UK

Transglutaminases (TGs; EC 2.3.2.13) are one of the most represented enzyme families that imply multiple functions. To date nine members of this family have been described in in different mammalian tissues: keratinocyte (TG1), tissue (TG2), epidermal (TG3), prostate (TG4), type 5 (TG5), neuronal (TG6), type 7 (TG7), blood coagulation factor XIII-A subunit (FXIII-A), and the catalytically inactive erythrocyte band 4.2 protein. The most studied member among them is TG2. It has several cellular localisations and participates in several physiological and pathological processes^1^. TG2 can exist in a GTP-bound signaling-active conformation or in a calcium dependent transamidase-active conformation. The role of TG2 in cancer was recently highlighted in different recent studies. In this presentation the authors will review TG2-regultated pathways that are involved in cancer development and progression in various tumor types. Recently the authors developed new application of the microbial transglutaminase in the field of Antibody Drug Conjugate ((ADC). CovIsoLink™ (Covalently Isopeptide crosslinking) relates to methods for enzymatic covalently coupling drugs and other compounds through transglutaminase site specific generated in the targeted proteins including, polypeptides, proteins and immunoglobulins (patent pending1). Monoclonal antibodies coupled to highly toxic agents or ADC (Antibody-Drug Conjugate) are becoming a significant component of anticancer treatment. ADCs are a new class of powerful drugs designed to target high-dose chemotherapy directly to cancer cells. Trastuzumab/emtansine (T-DM1 Kadcyla R) is an ADC that has been shown to cause significant. Despite their growing success, commercial ADCs are still heterogeneous mixtures. They are largely manufactured using chemical conjugation methods, in which the cytotoxic drug is covalently attached to lysines or cysteines on the antibody. These methods generally result various number of drugs (0 to 8) attached to different positions on the antibody determining the DAR (drug-antibody ratio). At present, the DAR distribution is not fully controlled, while it influences the pharmacokinetic-pharmacodynamic profiles of ADCs: the naked antibody (DAR0) is a competitive inhibitor, ADCs with a low DAR display poor efficiency, and those with a high DAR are rapidly eliminated in plasma. Depending on the payload, DAR2 seems to be the best compromises. CovIsoLink™ is used to develop new ADCs since the major advantage of this method is to obtain a homogenous immunoconjugate with uniform stoichiometry by controlling: (a) the location of coupling sites on the antibody without affecting its immunoreactivity and (b) the number of molecules coupled per molecule of antibody by controlling the DAR and consequently the toxicity and efficacy of therapeutic molecules^2^. The authors will discuss the results of our ADC in comparison with Trastuzumab-emtansine (T-DM1) targeting Her2/neu, using series of in vitro and in vivo models.

Disclosure: PCT/EP2014/0792278

References

1. Sivadó, É. et al. Optimised methods (SDS/PAGE and LC-MS) reveal deamidation in all examined transglutaminase-mediated reactions. FEBS Open Bio 9, 396–404 (2019). 10.1002/2211-5463.12575

2. Ager, C. et al. 31st Annual Meeting and Associated Programs of the Society for Immunotherapy of Cancer (SITC 2016): part two. J. Immunother. Cancer 4, 73 (2016). 10.1186/s40425-016-0173-6

## RPC 34

### TG2 as a potential marker for DNA damaging drug resistance

#### Evgenii Y Smirnov, Olga A Fedorova, Alexandra A Daks, Nick Barlev, Mauro Piacentini

##### Institute of Cytology RAS, St.-Petersburg, Russia

Tissue transglutaminase (TG2) is a multifunctional enzyme that is known for taking part in many aspects of cell functioning including adhesion, growth, migration, differentiation, apoptosis and many others. It was shown that it also takes part in drug resistance that is significant problem in medicine. In this study the authors investigated the potential molecular mechanism of TG2 involvement in drug resistance and DNA damage. To understand the role of TG2 in response to DNA damage the authors performed the MTT assay on TG2 knockout and wt MEF cell lines with and without doxorubicin treatment. The MTT assays showed that there is an increase in cell death in TG2 knockout cells in comparison to wild type. To understand the role of TG2 in apoptosis MEF cell lines (control and TG2 knockout) were treated with etoposide followed by annexin V staining. The amount of cells that underwent apoptosis was increased in TG2 knockout cells. Additionally, to study the potential molecular pathway the authors performed an immunoprecipitation to detect the interaction between TG2 and both γ-H2aX and HSF1 protein after DNA damage caused by doxorubicin treatment. here was also an increased interaction between TG2 and γ-H2aX under DNA damaging conditions. In sum, the absence of TG2 correlates with lowered cell resistance to DNA damage, that also correlates with TG2 and γ-H2aX interaction under DNA damaging conditions. However, in order to obtain a better understanding of the role of TG2 in DNA damage further investigation needs to be done.

Disclosure: The work was supported by the grant from the Russian Government Programme for the Recruitment of the leading scientists into the Russian Institutions of Higher Education 14.W03.31.0029 and Russian Science Foundation grant# 19-45-02011.

## RPC 35

### Correlation analysis between SDHx mutations and immunostaining of SDHB subunit in carotid paragangliomas

#### Anastasiya V Snezhkina^1^, Maria S Fedorova^1^, Dmitry VKalinin^2^, Vladislav S Pavlov^1^, Elena N Lukyanova^1^, Alexander L Golovyuk^2^, Elena A Pudova^1^, Maria V Savvateeva^1^, Oleg A Stepanov^1^, George S Krasnov^1^, Anna V Kudryavtseva^1^

##### ^1^Engelhardt Institute of Molecular Biology, Russian Academy of Sciences, Moscow, Russia; ^2^Vishnevsky Institute of Surgery, Ministry of Health of the Russian Federation, Moscow, Russia

Carotid paragangliomas (CPGLs) belong to rare head and neck tumors of neuroendocrine origin. CPGLs are often associated with mutations in the SDHx genes^1^. SDHx mutations are currently determined using immunohistochemistry (IHC) or targeted sequencing. Several reports suggested that SDHB immunostaining could reflect mutations in any SDHx genes^2^. In the study, the authors performed a correlation analysis between SDHx mutation status and SDHB immunoreactivity in CPGLs that is significant to improve genetic testing of the tumor and understanding of CPGL pathogenesis. Exome analysis (focused on SDHx) and immunohistochemistry study were performed for 42 CPGLs; present/absent mutations in the SDHx genes (SDHA, SDHB, SDHC, and SDHD) was compared with immunostaining of SDHB subunit. High-throughput exome sequencing and bioinformatic analysis were carried out as described in our previous works^3,4^. SDHB protein level was estimated with immunohistochemistry. Pathogenic/likely pathogenic mutations in SDHx genes were found in 20 out of 42 studied CPGLs (48%). In 17 from 20 CPGLs with SDHx mutations, negative or weak diffuse SDHB staining was revealed. Most samples carried SDHD mutation (13/20); weak diffuse staining of SDHB was detected in nine from them, and negative SDHB staining was observed in two cases. In two samples from thirteen with SDHD mutations, SDHB was immunopositive. Variants in the SDHB gene were found in three CPGLs; in two cases, SDHB was immunonegative, and the authors detected positive SDHB staining in a sample. SDHC mutations were also revealed in three CPGLs. All these samples were characterized by negative or weak diffuse staining of SDHB. In one CPGL, the authors observed pathogenic variant in the SDHA gene that occurs along with weak diffuse SDHB staining. In CPGLs without mutations in any SDHx genes (52%, 22/42), the authors also revealed negative or weak diffuse SDHB staining (9 from 22 cases). The obtained results indicate that SDHB immunostaining does not fully reflect the presence of mutations in the SDHx genes, and genetic testing is recommended for patients with CPGLs.

Disclosure: This work was supported by the Russian Science Foundation, grant no. 17-75-20105.

References

1. Snezhkina, A. V. et al. Exome analysis of carotid body tumor. BMC Med. Genomics 11, 17 (2018). 10.1186/s12920-018-0327-0

2. van Nederveen, F. H. et al. An immunohistochemical procedure to detect patients with paraganglioma and phaeochromocytoma with germline SDHB, SDHC, or SDHD gene mutations: a retrospective and prospective analysis. Lancet Oncol. 10, 764–771 (2009). 10.1016/S1470-2045(09)70164-0

3. Snezhkina, A. V. et al. Novel potential causative genes in carotid paragangliomas. BMC Med. Genet. 20, 48 (2019). 10.1186/s12881-019-0770-6

4. Kudryavtseva, A. V. et al. Mutational load in carotid body tumor. BMC Med. Genomics 12, 39 (2019). 10.1186/s12920-019-0483-x

## RPC 36

### Pyroptosis: A New Therapeutic Approach for Glioblastoma Therapy

#### Gulcin Tezcan^1^, Zarema E Gilazieva^1^, Albert A Rizvanov^1^, Svetlana F Khaiboullina^1,2^

##### ^1^Institute of Fundamental Medicine and Biology; Kazan Federal University, Kazan, Russian Federation; ^2^Department of Microbiology and Immunology, University of Nevada, Reno, NV, USA

Glioblastoma (GBM) is one of the most challenging malignancies in all of oncology. Despite of the significant progress made in multimodal therapy of GBM, these patients generally have a dismal prognosis with a short survival rate. This is often associated with the resistance to chemotherapeutic agents, including Temozolomide. Therefore there is an urgent need to develop alternative therapeutic approaches for these patients. Pyroptosis, a recently discovered form of inflammatory programmed cell death, is dependent on activation of Caspase-1 and release of inflammatory mediators, including IL-1β and IL-18. Pyroptosis is associated with identification and clearance of pathogens that by activating signaling pathways. Morphologically, it characterized by cell swelling and plasma membrane rupture. However, the mechanism of induction of pyroptosis-mediated cancer cell death remains largely unclear. The objective of this study was to investigate the role of inflammasome activation induced pyroptosis in GBM cell death. To stimulate pyroptosis, U138MG (GBM) cells were treated with Nigericin, a microbial toxin derived from Streptomyces hygroscopicus which acts as a potassium ionophore. The effect of Nigericin on stimulation of pyroptosis was analyzed by detection of Caspase-1 transcripts and protein secretion of IL-1β using RT-qPCR and ELISA respectively. Tumor cell vitality was determined using Annexin V, cell proliferation assay, LDH assay. Effect of pyroptosis on angiogenesis was demonstrated by tube formation assay. Statistical analysis was done using one-way ANOVA and Tukey’s analyses and Kruskal–Wallis one-way analysis of variance. Our results demonstrated that stimulation of pyroptosis decreased the cell viability, proliferation and angiogenesis in GBM cell line as compare to untreated control (p < 0.05). Our results suggest that, stimulation of pyroptosis has a potential for GBM treatment.

## RPC 37

### SAM68 protein is involved in the Apoptotic microtubule network (AMN) organization

#### Elena A Vasileva^1^, Viktoria A Mamontova^1^, Olga A Fedorova^1^, Alexandra A Daks^1^, Oleg Y Shuvalov^1^, Nickolai A Barlev^1^

##### ^1^Institute of Cytology RAS, Saint-Petersburg, Russia

Sam68 protein belongs to the STAR family (signal transduction and activation of RNA metabolism) of RNA-binding proteins. The importance of Sam68 was demonstrated in colon, breast, cervical and prostate cancers, kidney and esophagus carcinomas^1,2^. Sam68 is known to be involved in several stages of mRNA metabolism, regulation of cell cycle and apoptosis^3^. The authors have shown that the GFP-Sam68 protein forms microtubules-like structures in the cytoplasm of individual cells in HEK293T and HCT116 cell lines. Immunofluorescence staining of cells using anti-α-tubulin antibodies demonstrated that Sam68 is associated with microtubules. Microscopic examination of individual cells revealed defective micronucleus, cell rounding and decrease in cell size. The authors found that such microtubules are closely associated with the plasma membrane, forming a cortical ring. Cell staining with the TMRE reagent (Mitochondrial Membrane Potential Assay Kit) and MitoTracker™ Red CMXRos kits confirmed that Sam68 is associated with the formation of the apoptotic microtubule network (AMN).

Disclosure: The study was supported by Russian Fund for Basic Research grant No. 18-315-00408 mol_a and RSF grant 19-45-02011.

References

1. Liao, W.-T. et al. High expression level and nuclear localization of Sam68 are associated with progression and poor prognosis in colorectal cancer. BMC Gastroenterol. 13, 126 (2013). 10.1186/1471-230X-13-126.

2. Elliott, D. J. & Rajan, P. The role of the RNA-binding protein Sam68 in mammary tumourigenesis. J. Pathol. 222, 223–226 (2010). 10.1002/path.2753.

3. Liao, W.-T. et al. High expression level and nuclear localization of Sam68 are associated with progression and poor prognosis in colorectal cancer. BMC Gastroenterol. 13, 126 (2013). 10.1186/1471-2121-5-5.

## RPC 38

### Orphan Nuclear Receptor NR4A1 as a Potential Predictive Marker of Chemotherapy Resistance in Breast Cancer

#### Petr Yudichev

##### Institute of Cytology RAS, Saint-Petersburg, Russia

It has been shown that NR4A1 is involved in a myriad of cellular processes, including: cellular metabolism, proliferation, DNA damage repair, apoptosis and cell death, differentiation and embryogenesis. Aberrations in NR4A1 expression are well-documented in breast, pancreatic, testicular and lung cancers. Breast cancer is the most diagnosed cancer type in women, making up 25 % of total cases. One of the serious problems of cancer chemotherapy is multiple drugs resistance arising in heterogeneous cancer cell population. Possible mechanisms of multiple drug resistance include detoxification, drug efflux, resistance to apoptosis, aberrations in cell cycle control, DNA repair upregulation and EMT. Due to the spectrum of regulated processes and other non-genomic functions it has been suggested that NR4A1 may contribute to cancer cell ability to gain resistance to DNA-damaging agents, such as doxorubicin or cisplatin. Finding new molecular markers for chemotherapy resistance is an actual topic of research as it allows assessment of therapy regimen for patients with tumors expressing high levels of molecular markers. Our work was conducted on two different breast cancer cell lines with different NR4A1 expression levels, MDA-MB-231 and MCF-7. In this study the authors performed lentiviral transduction to knockdown NR4A1 with shRNA. Transduction with pLKO.1 coding nonspecific scrambled RNA was used to establish a control cell line. The authors treated those cell lines with different concentrations of DNA-damaging agents and further assayed cell viability with MTT and LDH tests. The authors have shown that control cell line exhibits higher survival rates. The authors then investigated possible mechanisms of NR4A1 action on heightened cell viability with FACS-analysis of apoptosis and cell cycle, RT-PCR for pro-apoptotic genes and western-blotting. Based on our findings and literature data the authors propose that NR4A1 may contribute to resistance to DNA-damaging agents in cancer cells and can be further investigated as a perspective novel marker of chemotherapeutic resistance.

Disclosure: This work was supported by Russian Science Foundation grant #18-75-10076.

## RPC 39

### Protective Effect of mTOR Inhibitor Rapamycin during Doxorubicin Treatment in Transgenic HER-2/neu Mice

#### Maria N Yurova, Ekaterina A Gubareva, Margarita L Tyndyk

##### Federal State Budget Institution «N.N. Petrov National Medical Research Centre of Oncology» Ministry of Public Health of Russian Federation

Female transgenic FVB/N mice carrying the breast cancer gene HER-2/neu with detected first mammary tumor (average tumor volume per group 0.2 ± 0.06 cm3) for 4 weeks were injected with rapamycin (RAP) 0.45 mg/kg every other day; doxorubicin (DOX) 1.87 mg/kg two times a week; or a combination thereof. Control animals were injected intraperitoneally every other day with 0.2 ml of solvent (2% ethanol solution). Under RAP treatment a tendency toward inhibition of tumor growth was observed, maximally by 34% (p > 0.05). Administration of DOX significantly inhibited the mammary tumors development by 46–62% (p < 0.05 since 14 day of experiment). The addition of RAP to DOX therapy enhanced tumor growth inhibition to 52-78% (p < 0.05 since 14 day of experiment). The level of proliferative activity (EdU, IHC staining with antibodies to PCNA) was evaluated in tumor and healthy tissues. There was no difference in the number of proliferating cells in jejunum crypts of treated animals in comparison with control. A statistically significant decrease in the level of proliferation was observed in the tumor tissue: in the group treated with RAP by 30%; DOX—by 40%, the combination of DOX + RAP—by 50% (p < 0.05). The protection of healthy tissues from the toxic effects of chemotherapy was determined by the level of apoptosis in jejunum crypts (morphological study) and the expression level of the gammaH2AX DNA damage marker in liver (IHC staining with anti-gammaH2AX antibody). Analysis of apoptosis in jejunum crypts in female HER-2/neu mice did not reveal the toxic effect of RAP therapy. DOX therapy had a pronounced toxic effect, whereas the addition of RAP abated DOX toxicity effect. In liver cells, the addition of RAP to DOX therapy significantly (p < 0.05) reduced the level of gammaH2AX expression. Thus, the addition of RAP to DOX treatment enhanced the antitumor effect of the chemotherapeutic drug and showed a protective effect on healthy tissues (jejunum, liver) in murine model of spontaneous mammary tumors with overexpression of HER-2/neu gene.

Disclosure: This study was supported by Russian Science Foundation (No. 17-75-10112).

